# How Pyroptosis Contributes to Inflammation and Fibroblast-Macrophage Cross-Talk in Rheumatoid Arthritis

**DOI:** 10.3390/cells11081307

**Published:** 2022-04-12

**Authors:** Benjamin Demarco, Sara Danielli, Fabian A. Fischer, Jelena S. Bezbradica

**Affiliations:** The Kennedy Institute of Rheumatology, University of Oxford, Oxford OX3 7FY, UK; sara.danielli@kennedy.ox.ac.uk (S.D.); fabian.fischer@linacre.ox.ac.uk (F.A.F.)

**Keywords:** inflammation, pyroptosis, inflammasomes, gasdermins, rheumatoid arthritis, fibroblast–macrophage cross-talk, TAM receptors

## Abstract

About thirty years ago, a new form of pro-inflammatory lytic cell death was observed and termed pyroptosis. Only in 2015, gasdermins were defined as molecules that create pores at the plasma membrane and drive pyroptosis. Today, we know that gasdermin-mediated death is an important antimicrobial defence mechanism in bacteria, yeast and mammals as it destroys the intracellular niche for pathogen replication. However, excessive and uncontrolled cell death also contributes to immunopathology in several chronic inflammatory diseases, including arthritis. In this review, we discuss recent findings where pyroptosis contributes to tissue damage and inflammation with a main focus on injury-induced and autoimmune arthritis. We also review novel functions and regulatory mechanisms of the pyroptotic executors gasdermins. Finally, we discuss possible models of how pyroptosis may contribute to the cross-talk between fibroblast and macrophages, and also how this cross-talk may regulate inflammation by modulating inflammasome activation and pyroptosis induction.

## 1. Pyroptosis and Inflammasomes

### 1.1. Discovery of Pyroptosis and Inflammasomes

Back in the 1990s, several studies described a peculiar type of cell death, initially categorised as apoptosis, that was accompanied by mature interleukin-(IL)-1β release in macrophages infected with *Shigella flexneri* (*S. flexneri*) and *Salmonella enterica* serovar *Typhimurium* (*S. Typhimurium*) [1,2,3]. Further experiments challenged this cell death categorization by observing that the non-apoptotic enzyme caspase-1 was indispensable for the detected pro-IL-1β processing, and that the cellular morphology of *S*. *Typhimurium*-infected cells, such as the loss of membrane integrity, differed from the morphology of cells stimulated with classic apoptosis inducers [4,5,6,7,8]. This new characterized cell death form was named ‘pyroptosis’ derived from the Greek ‘*pyro*’ (fire, fever) and ‘*ptosis*’ (falling) to accentuate its inflammatory characteristics [9].

The mechanism of caspase-1 activation during pyroptosis was only cleared in 2002, when it was demonstrated that caspase-1 is activated through the formation of multiprotein complexes termed as ‘inflammasomes’ [10]. The inflammasome in this study was composed of the sensor, pattern recognition receptor (PRR) called nucleotide-binding oligomerization domain, leucine-rich repeat and pyrin domain containing (NLRP) 1, the adapter, apoptosis-associated speck-like protein containing a CARD (ASC), and the effector enzyme, caspase-1. When inflammasomes are formed, the pyrin domain of ASC allows its interaction with the pyrin domain of NLRP1, while the CARD domain of ASC allows its interaction with the CARD domain of caspase-1 promoting caspase-1 recruitment to the signalling complex and its activation [11,12]. Active caspase-1 cleaves pro-inflammatory cytokines such as the inactive precursor pro-IL-1β in its biologically active form (referred as IL-1β in the text).

Following these early studies, contributions from many laboratories defined new inflammasomes and allowed a better understanding of their biology (detailed review of inflammasomes and their regulation in [13,14,15,16]). In the next section, we mainly discuss the NLRP3 inflammasome and its mechanisms of regulation, and we briefly discuss latest findings regarding other inflammasomes.

### 1.2. NLRP3 Inflammasome Regulation and Activation

The best-characterised inflammasome is the NLRP3 inflammasome, which was briefly discovered after the NLRP1 inflammasome [17]. NLRP3 activation starts with the so-called inflammasome ‘priming’ or ‘signal 1’. During the priming phase, pathogen- and damage-associated molecular patterns (PAMPs/DAMPs) such as lipopolysaccharide (LPS) or inflammatory cytokines such as tumour necrosis factor (TNF) induce the upregulation of NLRP3 and pro-IL-1β via the nuclear factor-κB (NF-κB) pathway [18,19,20]. Interestingly, the nature of signal 1 dictates how rapidly inflammasome activation occurs [21]. Macrophages stimulated with microbial signals activate inflammasomes with faster kinetics than when sterile signals are used, suggesting that cells are more prone to react upon infections, when a rapid inflammatory response is needed. Components of other cell death signalling pathways, for example, caspase-8, known to drive apoptosis, also regulate inflammasome priming [22,23,24,25,26]. Mechanistically, caspase-8 promotes full transcriptional induction of pro-IL-1β and cleaves the suppressor of pro-inflammatory cytokine production called NEDD4-binding protein 1 [27,28]. While it was first believed that inflammasome priming is indispensable for NLRP3 activation, emerging studies have also demonstrated that inflammasomes can be activated when signal 1 and signal 2 are added simultaneously, and even without signal 1 priming in some cases (reviewed in detail in [29]). Many studies have shown that this transcriptionally independent NLRP3 inflammasome regulation relies on post-translational modifications (PTMs) such as phosphorylation and ubiquitination events (the regulation of NLRP3 inflammasome by PTMs is reviewed in detail in [16,30]).

‘Signal 2’ is referred to the stimulation signal that leads to the assembly of the inflammasome complex and its activation. It is typically a signal that indicates the presence of pathogen invasion or excessive tissue damage and generally causes the loss of cellular homeostasis. Remarkably, back in 1994 and prior to the inflammasome discovery, it was already observed that the bacterial ionophore toxin nigericin, or high adenosine triphosphate (ATP) extracellular levels, induced the processing and release of active IL-1β in macrophages, which was blocked by increasing concentrations of extracellular potassium [31,32,33]. Later studies confirmed that NLRP3 inflammasome senses loss of intracellular potassium upon stimulation with various signals 2, such as the toxin nigericin, extracellular ATP or extracellular insoluble crystals [34,35]. How NLRP3 inflammasome senses potassium efflux is still unknown, but one recently proposed model is that the loss of intracellular potassium promotes NLRP3 inflammasome activation by triggering a structural change of NLRP3 into its active conformation [36]. While it was accepted that potassium efflux is a common trigger of NLRP3 inflammasome, other studies have shown that exceptions exist, and that macrophages stimulated with imiquimod, N-acetylglucosamine, or human monocytes treated with LPS can activate NLRP3 inflammasome in a potassium efflux-independent manner [37,38,39]. Since early potassium-efflux blocking experiments were performed using potassium chloride, it was speculated that changes in chloride concentration levels could contribute to NLRP3 inflammasome activation. Indeed, several later reports confirmed that chloride efflux, through multiple chloride channels, also promotes NLRP3 inflammasome assembly [40,41,42,43]. 

In which location in the cell the NLRP3 inflammasome is activated has also been intensively investigated and could be a key to understanding inflammasome regulation. Recent studies suggested that the NLRP3 is recruited to dispersed trans-Golgi network (TGN) [44] and that NLRP3 inflammasome assembly occurs at the microtubule-organizing centre [45]. Others proposed that NLRP3 senses disrupted endosomal trafficking [46], but all generally support the idea that NLRP3 somehow senses intracellular organelle dysfunction (reviewed in detail in [47]). In healthy cells, inactive full-length NLRP3 forms a double-ring cage of 12–16 monomers located at various membrane organelles [48,49]. Within this cage, the pyrin domain is shielded to prevent its premature association with ASC and spontaneous inflammasome activation. Despite the contributions of many laboratories to define how the NLRP3 inflammasome activity is controlled, and on which organelle each activation step occurs, there are still open questions to be addressed to achieve a complete understanding of the mechanisms by which the NLRP3 inflammasome can detect such a wide variety of compounds.

### 1.3. Other Inflammasomes

In addition to the NLRP3 inflammasome, there are other inflammasomes composed of different cytosolic PRRs which allow cells to detect diverse PAMPs/DAMPs. It is now appreciated that, like NLRP3, several of them also contribute to sterile inflammation. In this context, we briefly highlight the latest discoveries of the NLRP1, NLR family CARD domain-containing protein 4 (NLRC4), absent in melanoma 2 (AIM2) and pyrin inflammasomes, and the non-canonical inflammasome pathway activation (detailed reviews focused on inflammasomes have been extensively published in recent years [13,14,15,50,51,52,53,54,55,56]).

Activating germline mutations in NLRP1 inflammasome cause chronic inflammatory skin disorders in humans [57,58,59,60]. One of the activation mechanisms of murine NLRP1B (mice express 3 NLRP1 isoforms) was mapped during infection and occurs upon the cleavage of its N-terminus by bacteria-derived enzymes [61,62]. The exposed N-terminal domain results in proteasome-mediated degradation and N-terminal processing of NLRP1B, releasing the C-terminal domain, which acts as a caspase-1 activator. Exceptions to this model exist, as NLRP1B is also activated upon *Toxoplasma gondii* infection, where the N-terminal processing of NLRP1B is not observed [63]. Human NLRP1 is similarly activated upon pathogen-induced cleavage, in this case by viral enzymes [64,65]. In keratinocytes, human NLRP1 is also activated upon ultraviolet B (UVB) radiation [66,67], where it senses UVB-triggered ribotoxic stress [68,69]. Mice express low-to-undetectable protein levels of NLRP1 in the skin [67], suggesting a unique role of NLRP1 in humans. Highlighting another difference between species, human NLRP1, but not murine NLRP1B, recognizes double-strand RNA, revealing that NLRP1 is also a sensor of virus-associated nucleic acids [70]. Recently, two preprint studies have shown that the NLRP1 inflammasome can be activated upon abnormal accumulation of cytosolic peptides, and upon cytosolic reductive stress, highlighting NLRP1 as a possible broad sensor of cytoplasmic dysfunction [71,72].

Mutations in NLRC4 cause autoinflammatory diseases [73,74], highlighting the role of this inflammasome in sterile inflammation as well. The mechanism of activation for NLRP4 was also mapped during infection. The NLRC4 inflammasome recognizes cytosolic bacterial proteins derived from the type-3 secretion system, and flagellin via accessory proteins called NLR family apoptosis inhibitor proteins (NAIPs). The NLRC4 inflammasome has been recently reviewed in detail in [75,76]; here, we briefly highlight only a few of the latest findings suggesting the possibility that NLRC4 may be activated in sterile inflammation. The phospholipid lysophosphatidylcholine, known to be accumulated in pathological neurological diseases and cause neuroinflammation, induces NLRC4 and NLRP3 inflammasome activation in microglia and astrocytes [77], and so does hyperosmotic stress [78]. Another role of the NLRC4 inflammasome during sterile inflammation was revealed in a recent study, where endogenous retroelements called short interspersed nuclear element (SINE) encoded RNAs were detected by the DEAD-box helicase 17 to trigger NLRC4 inflammasome activation independently of NAIPs [79]. These studies highlight a novel function of NLRC4 inflammasome in sterile inflammation and may have broad implication as SINE RNAs have been associated with multiple immune-mediated diseases [80].

The AIM2 inflammasome typically detects the cytosolic presence of DNA as a signal of bacterial and viral infection (the AIM2 inflammasome was recently reviewed in detail in [52,53,54]). However, its role is not limited to infection, as AIM2 can also become activated by self-DNA, for example, upon DNA-damage during chemotherapy, upon loss of nuclear envelope integrity, upon loss of mitochondrial integrity, or as a result of self-DNA accumulation in the cytosol, when cytosolic nucleases such as DNAse II are deficient. In arthritis models, the AIM2 inflammasome is reported to contribute to joint inflammation in a model of chronic polyarthritis, where AIM2 detects self-DNA [81,82].

The pyrin inflammasome is encoded by the human *MEFV* gene, and mutations in this gene cause systemic autoinflammatory syndromes as well [83,84,85]. In healthy cells, pyrin is kept inactive by binding to 14-3-3 proteins. This is possible because the Rho GTPase RhoA recruits kinases that phosphorylate pyrin to allow its biding to 14-3-3. If Rho GTPases become inactivated by bacterial toxins as a part of the pathogen immune evasion strategy, pyrin is no longer phosphorylated; it dissociates from 14-3-3 and forms an inflammasome (the pyrin inflammasome was recently reviewed in detail in [55,56]). Thus, just like NLRP3, pyrin is activated indirectly upon the loss of cellular homeostasis.

The non-canonical inflammasome pathway is activated when LPS from Gram-negative bacteria reaches the cellular cytoplasm and triggers murine caspase-11 activation (caspase-4/5 in human) [86,87]. Caspase-11 can also be activated during sterile inflammation by detecting oxidised membrane phospholipids from dying cells [88]. It has been demonstrated by different laboratories that LPS recognition by caspase-11 is regulated by interferon-inducible guanylate-binding proteins (GBPs) (reviewed in detail in [51,89]). GBP1 is first recruited to intracellular pathogens, which promotes the recruitment of other GBPs to build an assembly platform that triggers caspase-11 activation [90,91]. Other reports have also identified other regulators of the non-canonical inflammasome pathway. For instance, the cytokine receptor-associated TYK2 promotes it [92], while interferon (IFN)-inducible Irgm2 and Gate-16 act as negative regulators [93,94,95], suggesting that IFN-driven priming of the non-canonical inflammasome is likely followed by a negative feedback loop to prevent excessive inflammation and immunopathology. Interestingly, a preprint suggests a role of NLRP11 as an upstream sensor of cytosolic LPS that drives caspase-4 activation in human macrophages [96]. How oxidised membrane phospholipids are directly detected by caspase-11 is less well understood.

Independent of which inflammasome is assembled, caspase-1 activation is the common downstream event. However, the mechanisms by which caspase-1 activation induces IL-1β release, a cytokine known to lack a secretion signal [97], and triggers pyroptosis were unclear until the discovery of the gasdermin family as the pyroptotic executors.

## 2. Gasdermins

### 2.1. Gasdermin Family

Back in 2000, a gene located in the mouse chromosome 11 responsible for causing abnormal skin and hair development was mapped and termed ‘gasdermin’ (now known as gasdermin A1) due to its expression pattern in the upper gastrointestinal tract and dermis [98,99]. The gasdermin protein family in humans is composed by GSDMA (three isoforms in mice, GSDMA1–3), GSDMB (not expressed in mice), GSDMC (four isoforms in mice, GSDMC1–4), GSDMD, GSDME, and pejvakin (PJVK). Gasdermins (GSDMs) share common N- and C-terminal domains (with the exception of PJVK that has a truncated C-terminal domain), which are connected via a less-conserved linker region [98]. Two recent reviews show a complete overview of each GSDM expression in human tissue (Figure 1 in [100], and Figure 2 in [101]). Evolutionary analysis demonstrated that GSDME and PJVK appeared first from a common ancestor, following a gene duplication event. Then, GSDMA (gene duplication from GSDME) appeared, followed by the other GSDMs [101,102]. GSDMs are not only found in mammals. Corals and other invertebrate species have a functional GSDME involved in pathogen-induced necrotic death [103]. Recently, 50 bacterial gasdermins (bGSDMs) were revealed by sequence analysis of bacterial antiphage defence islands [104]. In this study, a shorter C-terminal domain was observed in bGSDMs compared to mammalian GSDMs. Fungal gasdermin-like proteins, which are involved in allorecognition-induced cell death, have also been recently discovered [105]. Interestingly, the fungal gasdermin-like protein studied (RCD-1) does not harbour a C-terminal domain, as observed in mammalian GSDMs. Further in silico analysis of genomic landscapes showed that gasdermin genes cluster with protease-encoding genes, suggesting that these proteins are activated by cleaving events [106].

### 2.2. Gasdermins as Pyroptotic Executors

**Figure 1:**
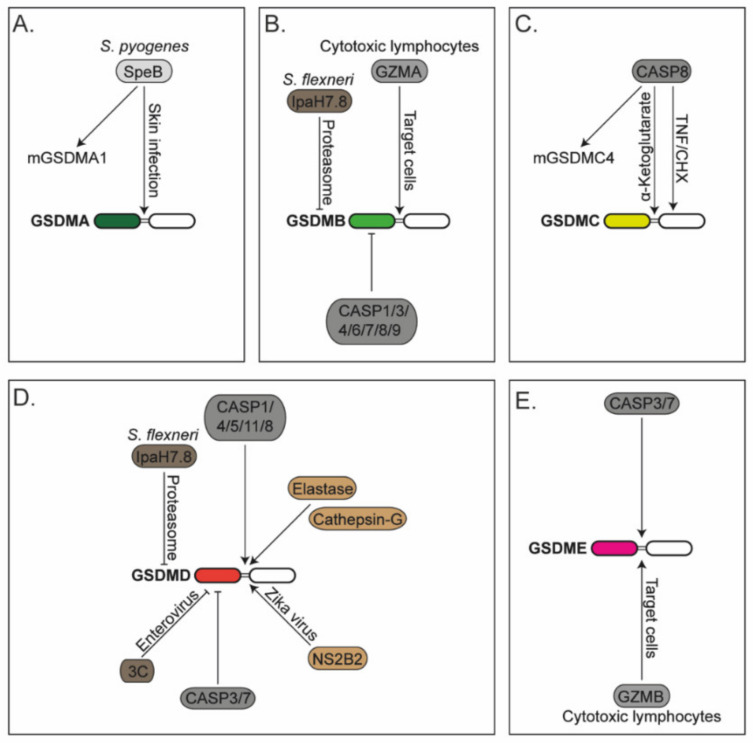
Regulation of gasdermins: (**A**) Human gasdermin-A (GSDMA) and murine GSDMA1 are cleaved by the cysteine protease SpeB from *Streptococcus pyogenes* (*S. pyogenes*) during skin infection. (**B**) Gasdermin-B (GSDMB) is cleaved by granzyme-A (GZMA) delivered by cytotoxic lymphocytes. GSDMB is cleaved and inactivated by recombinant caspases-1/3/4/6/7/8/9 (CASP1/3/4/6/7/8/9). IpaH7.8 effector protein from *Shigella flexneri* (*S. flexneri*) ubiquitinates GSDMB that triggers its degradation via the proteasomal pathway. (**C**) Human gasdermin-C (GSDMC) and murine GSDMC4 are cleaved by caspase-8 (CASP8) in cells treated with tumour necrosis factor (TNF) and cycloheximide (CHX) or cells stimulated with α-ketoglutarate. GSDMC is cleaved at two different sites by CASP8. (**D**) Gasdermin-D (GSDMD) is cleaved by caspases-1/4/5/11/8 (CASP1/4/5/11/8), neutrophil protease elastase, cathepsin-G and NS2B2 from Zika virus. GSDMD is cleaved and inactivated by caspases-3/7 (CASP3/7) and the viral protease 3C from enteroviruses. The IpaH7.8 effector protein from *S. flexneri* ubiquitinates GSDMD that triggers its degradation via the proteasomal pathway. (**E**) Gasdermin-E is cleaved by CASP3/7 and granzyme-B (GZMB) derived by cytotoxic lymphocytes.

Soon after the discovery of the gasdermin family, these proteins were associated with cell death in yeast and mammalian cells [107,108]. The mechanisms by which GSDMs trigger cell death was unclear until 2015, when two landmark studies revealed GSDMD as the executor of the lytic cell death pyroptosis [109,110]. First, it was shown that cells lacking GSDMD have impaired lytic cell death and IL-1β secretion after LPS transfection. Murine caspase-11 directly cleaves GSDMD after the aspartic acid at position 276 located in its linker region (Figure 1), a cleavage event that induces pyroptosis, and subsequent pro-IL-1β processing by caspase-1 upon secondary NLRP3 inflammasome activation (caspase-11 cannot cleave pro-IL-1β) [109]. Remarkably, and similar to the phenotype in *Casp11^−/−^* mice, *Gsdmd^−/−^* mice showed protection in a LPS endotoxic-shock model. In parallel, another study demonstrated that GSDMD is required for pyroptosis and IL-1β release after diverse pyroptotic stimuli, and caspases-1/4/5 can also cleave GSDMD at the same site as caspase-11 (Figure 1) [110]. A few months later, a third article came to the same results by performing high-sensitive quantitative mass spectrometry analysis [111]. Overall, these studies showed that GSDMD processing in its linker region liberates the N-terminal domain (GSDMD^NT^) from its autoinhibition by the C-terminal domain (GSDMD^CT^), which is required for pyroptosis. Interestingly, caspases-1/4/11 harbour an exosite that binds to GSDMD^CT^, which enhance the affinity for binding to GSDMD [112,113].

The discovery of GSDMD as a pyroptotic executor and its activation by pro-inflammatory caspases-1/4/5/11 gained the attention of many laboratories worldwide, which is reflected by the increase in publications in the following years. Already in 2016, four studies delivered key findings regarding the mechanisms by which GSDMD triggers pyroptosis [114,115,116,117]. Using different methods, such as electron microscopy, fluorophore-filled liposomes, and atomic force microscopy, these studies revealed that after GSDMD cleavage in its linker region, GSDMD^NT^ binds to acidic phospholipids and cardiolipin, forming pores on liposomes. These pores are large ring-shaped oligomers that are thermodynamically stable [118]. A recent publication showed that GSDMD pores are not constitutively open, but they are dynamic structures showing open–close states regulated by local phosphoinositide metabolism [119]. When GSDMD pores form, small intracellular molecules such as cleaved IL-1β can be released. Bigger proteins (>50 kDa) such as lactate dehydrogenase (LDH, a standard marker to measure lytic cell death) or high mobility group protein-1 (HMGB1) complexes can only be detected in the supernatant after cell lysis [120,121]. The release of molecules through GSDMD pores is not only regulated by the molecular size of the cargo but also by its electric charge [122,123]. The revealed GSDMD pore structure showed that the pore conduit is negatively charged with a 31- to 34-fold symmetry and an inner diameter of 21.5 nm in liposomes; thus, positively charged and neutral cargos are released in faster kinetics compared to negatively charged cargos with comparable molecular sizes [122]. In case of pro-IL-1β, its processing by caspase-1 exposes a polybasic charged patch that promotes membrane targeting and subsequent release [124]. Interestingly, the structure of GSDMA3 pores shows a 26- to 28-fold symmetry with an internal diameter of 18 nm [125], highlighting some variability in pore structure and size throughout GSDMs family members. Despite these differences in pore size, the overexpression of the N-terminal domain of GSDMA/B/C/E also induces lytic cell death in human cells [115]. PJVK, on the other hand, has lost its pore-forming activity, since it lacks key residues required for oligomerization and pore formation [102].

How GSDM pore formation leads to lytic cell death, and whether other factors are required, was still unclear, until a recent study revealed that the nerve injury-induced protein 1 (NINJ1) is indispensable for plasma membrane rupture (PMR) [126]. NINJ1 oligomerizes in response to cell death induction, and it is located at the plasma membrane, where it forms several speck-like assemblies. The NINJ1 requirement for plasma membrane rupture is not limited to pyroptosis; it was also observed upon necrosis and apoptosis induction. Interestingly, NINJ1-lacking cells showed a modest defect in cell lysis when necroptosis was triggered, suggesting that, different to GSDMD pores during pyroptosis, mixed lineage kinase domain-like protein (MLKL), known to be required for cell lysis during necroptosis, is sufficient to drive PMR. Interestingly, it has been recently proposed that the cytoprotecting effects of glycine, used for years to avoid terminal cell lysis, could be caused by a disruption of NINJ1 oligomerization when glycine is present [127]. The mechanisms by which NINJ1 induces lytic cell death are still unknown, but it is conceivable that NINJ1 could form large pores at the plasma membranes that would induce PMR. Regarding the in vivo role of NINJ1, *Ninj1^−/−^* mice are more susceptible to *Citrobacter rodentium* infection compared to WT mice, while they showed similar lethality in LPS-induced endotoxic-shock [126]. Interestingly, and before this publication, it has also been shown that *Ninj1^−/−^* mice are protected from susceptibility against experimental autoimmune encephalomyelitis due to reduced recruitment of leukocytes into the spinal cord, and these mice show milder pathology in a model of pulmonary fibrosis [128]. *Ninj1^−/−^* mice also had reduced levels of pro-inflammatory and pro-fibrotic mediators in the lungs compared to WT mice [129]. Whether the protective effects in these models were due to dysregulated PMR, and thus less DAMPs release in *Ninj1^−/−^* cells, requires further investigation. With the discovery of NINJ1 as the last executor of PMR, it is possible to investigate whether PMR and the release of large DAMPs, or the formation of GSDMD pores only together with a release of specific intracellular molecules (in cells lacking NINJ1), regulate host defence upon infections or inflammation in diseases.

Since GSDMs are required for pyroptotic cell death, the original definition of pyroptosis has been redefined as a ‘gasdermin-dependent type of necrotic cell death’ [130,131]. However, in some specific cases, this categorization might lead to confusion in the field; for instance, neutrophil NETosis, which has been shown to be GSDMD-dependent [132,133,134], is not categorized as pyroptosis.

### 2.3. Regulation of Gasdermin-D

Since GSDMD-driven pyroptosis leads to lytic cell death and the release of intracellular content, cells need to regulate this pathway to avoid unnecessary inflammation and tissue damage. Here, we discuss mechanisms that modulate GSDMD expression and activity, and how this affects pyroptotic levels.

#### 2.3.1. Transcriptional Levels

The first intrinsic mechanism that cells use to regulate pyroptosis and subsequent inflammation is to regulate the expression of GSDMs. GSDMD transcription is regulated by the interferon regulatory factor (IRF) 2, an effect that is partially compensated by IRF1 when IRF2 is absent [135]. Another study observed IRF1/2-dependent changes in GSDMD expression when cells were primed with IFN-γ; however, these effects were not detected in naïve cells [136]. Interestingly, the authors found that IRF1/2 also regulate caspase-4 expression levels. Overall, these findings show that IRF1/2 control inflammasome-related gene expression, and that GSDMD is transcriptionally regulated in certain circumstances. The GSDMs gene expression can also be regulated by epigenetic mechanisms. Type-I IFN, produced by macrophages infected with *Acinetobacter baumannii*, induces histone modifications that modulate *Gsdmd* gene expression [137]. Other transcriptional and epigenetic mechanisms that regulate GSDMs are extensively reviewed in [100].

#### 2.3.2. Post-Translational Modifications

GSDMD pore-forming activity may also be regulated by PTMs such as ubiquitination events. The E3 ligase synoviolin was shown to promote K27-linked polyubiquitination of GSDMD^NT^, which promotes pyroptosis [138]. Similarly, it has been shown that GSDME and GSDMA are regulated by phosphorylation events at threonine amino acids at positions 6 and 8, respectively, albeit phosphorylation in this region has not been reported for GSDMD [139]. Emerging studies have revealed the existence of other modifications in GSDMD and GSDME that regulate pyroptosis such as succination, itaconation, palmitoylation and oxidation [140,141,142,143,144].

#### 2.3.3. GSDMD Stability and Pore-Forming Activity

Not only the GSDMD expression but also its stability and pore-forming activity might be controlled to regulate pyroptosis. It was recently proposed that TRIM21 stabilizes protein levels of GSDMD [145], and a recent report also associated the mTOR Complex 1 (mTORC1), typically involved in metabolism [146], with GSDMD-driven pyroptosis [147]. Mechanistically, mTORC1 promotes mitochondrial reactive oxygen production, which promotes GSDMD^NT^ oligomerization, pore formation and pyroptosis.

#### 2.3.4. GSDMD Regulation by Apoptotic Caspases

Since the cytotoxic pore-function of GSDMs relies on the N-terminal fragment, its activity can be downregulated by proteolytic cleavage within this domain (Figure 1). Human GSDMD harbours a caspase-3/7 cleavage site on an aspartic acid (D) at the amino acid position 87 (D88 in mice) [148]. Murine macrophages expressing a caspase-3/7 cleavage-resistant GSDMD variant showed enhanced lytic cell death compared to WT cells, where GSDMD was partially inactivated by caspase-3/7 cleavage upon caspase-8-driven extrinsic apoptosis [149]. Remarkably, caspase-8 has a dual function, likely dependent on context. It can activate GSDMD directly, by cleaving in its linker region at the same site as caspases-1/4/5/11 [149,150,151,152], while in parallel, it can activate caspases-3/7 to terminate GSDMD activity by cleaving its N-terminus (Figure 1).

#### 2.3.5. Removal of GSDMD Pores

Regulating the number of pores formed at the plasma membrane also controls the kinetics and magnitude of pyroptosis. It was previously demonstrated that the endosomal sorting complexes required for transport (ESCRT) machinery can repair plasma membrane damage [153] and perforations in endolysosomes [154]. Furthermore, the ESCRT machinery downregulates cell death levels by facilitating the shedding of MLKL-damaged plasma membrane fragments during necroptosis [155]. A similar mechanism was shown to occur in pyroptotic cells, where the ESCRT machinery is recruited to the plasma membrane to repair/remove GSDMD pores and downregulate lytic cell death and pro-inflammatory cytokine release [156].

#### 2.3.6. Other, Non-Caspase Regulators

Not many years ago, it was believed that GSDMD was mainly activated by pro-inflammatory caspases-1/4/5/11. Later, laboratories independently demonstrated that apoptotic caspases-8/3/7 also regulate GSDMD activation, and this has now been expanded to non-caspase-related proteases (Figure 1). For instance, neutrophil protease elastase and cathepsin-G also activate GSDMD by cleaving within its linker region [133,134,157]. Similarly, the NS2B2 protease from Zika virus directly activates human GSDMD by cleaving at the amino acid arginine at position 249 to promote pyroptosis [158]. Similar to caspases-3/7, the viral protease 3C inactivates GSDMD by cleaving within GSDMD^NT^ [159]. Finally, it was recently reported that the nucleocapsid from SARS-CoV-2 associates with the GSDMD linker region to inhibit GSDMD cleavage and activation in human monocytes [160].

### 2.4. Regulation of Other Gasdermins

How the other GSDMs (GSDMA-E) are activated or inactivated has also been intensively investigated. Here, we provide a brief overview of the recently discovered mechanisms involving GSDMs regulation and highlight functions of full-length GSDMs. GSDMs have also been extensively reviewed in [161].

#### 2.4.1. GSMDA

It is well established that GSMDA^NT^ triggers pyroptosis in cells; however, its activation mechanism and physiological relevance has remained obscure for many years. A recent study demonstrated that human GSDMA and murine GSDMA1 are proteolytically activated by the cysteine protease SpeB after infection with the skin pathogen *Streptococcus pyogenes* (Figure 1) [162]. Cleaved GSDMA triggers pyroptosis in infected cells, which is beneficial for the host to control pathogen dissemination. Further studies are still required to reveal whether GSDMA activation occurs upon infection with other pathogens and to investigate whether and when the other murine GSDMAs (GSDMA2/3) trigger pyroptosis.

#### 2.4.2. GSMDB

GSDMB is activated upon direct cleavage by granzyme-A (Figure 1), delivered by cytotoxic lymphocytes, to induce its activation and pyroptosis of the target cell [163]. It can be inactivated by caspases-1/3/4/6/7/8/9, at least in experiments using overexpressed proteins and recombinant caspases [164,165]. GSDMB can also be inactivated by disrupting protein levels in cells. It is degraded in the proteasome following its ubiquitination by the IpaH7.8 effector protein from *S. flexneri* [166]. Interestingly, active GSDMB targets phospholipids found on Gram-negative bacterial membranes, and not the plasma membrane of the host cell, revealing microbiocidal activity to counteract pathogens and explaining why pathogens may have evolved effectors to induce the degradation of GSDMB. The same degradation mechanism occurs for human GSDMD (but not murine GSDMD) [167]. The ubiquitination and subsequent protein degradation rely on the first N-terminal amino acids of GSDMD, which differs between human and mouse. The reason why the first study observed degradation of GSDMB and not degradation of GSDMD [166] might be explained by the epitope tags located at the N-terminus of GSDMD used in the experiments, since this region is key for the IpaH7.8-mediated ubiquitination, and subsequent proteasomal degradation [167].

#### 2.4.3. GSMDC

Like GSDMD, GSDMC activation has been associated with apoptotic caspases. Recent studies have shown that caspase-8 activates human GSDMC by cleaving it in its linker region to promote pyroptosis (Figure 1) [168,169]. GSDMC can also be cleaved by caspase-6; however, it is still unknown in which context this occurs [168]. While in both studies the generated GSDMC^NT^ fragment induced pyroptosis, the identified cleavage site by caspase-8 was different. In cells treated with TNF and cycloheximide (CHX) under hypoxic conditions, GSDMC was processed by caspase-8 at the D365 residue [168], while in cells treated with α-ketoglutarate, the cleavage occurred at D240 [169]. How the same caspase discriminates where to cleave GSDMC in different circumstances is unknown. Humans only express one GSDMC, while mice have four isoforms (GSDMC1–4). Of those, only GSDMC4, but not the others, is cleaved by caspase-8 [169]. A recent report showed that the overexpression of full-length mouse GSDMC2 in HEK293 cells triggers pyroptosis; however, the activation mechanism is still unknown [170]. A preprint study showed that GSDMC2/3 are upregulated by IFN-λ and activated in the small intestine of irradiated mice [171]. Collectively, these data suggest that multiple GSDMC isoforms in mice may be able to induce cell death, but the enzyme that proteolytically activates them to release the pore-forming fragment may be different. Whether molecules can also be released through GSDMC pores was unknown until a recent study demonstrated that GSDMC pores allow IL-33 secretion, which drives host defence and intestinal inflammation in helminth infection [172].

#### 2.4.4. GSMDE

GSDME is processed into its active pore-forming fragment by apoptotic caspases-3/7 [173,174], the same ones that inhibit GSDMD [148,149]. GSDME is also activated by granzyme-B delivered by cytotoxic lymphocytes into GSDME-positive tumours (Figure 1) [175]. Remarkably, *Gsdme^−/−^* mice showed protection against chemotherapy-induced tissue damage and weight loss compared to WT controls when treated with the chemotherapeutical drug cisplatin, known to activate caspase-3 [173]. These mice are also protected from cytokine release syndrome (CRS), a serious side effect observed in chimeric antigen receptor (CAR) T cell therapy [176]. CRS is initiated when caspase-3 becomes activated in cancer cells via granzyme-B delivered from the CAR T cells. Active caspase-3 drives pyroptosis of GSDME-positive tumour cells and release of alarmins that activate caspase-1 and GSDMD in neighbouring macrophages, finally resulting in macrophage-driven release of pro-inflammatory cytokines and CRS. *Gsdme^−/−^* mice also have reduced chronic intestinal inflammation caused by epithelial-cell pyroptosis in a model of Crohn’s disease [177]. However, GSDME activity is not always pathological. During bacterial infection, GSDME allows the release of mature IL-1β from neutrophils in vitro and is beneficial for host defence against *Y. pseudotuberculosis* infection in vivo [178]. Similarly, GSDME is required for IL-1α release, pyroptosis and antiviral activity in keratinocytes and human skin organoids infected with vesicular stomatitis virus [179]. The same study shows that infection with herpes simplex virus 1 causes GSDME activation when the virus lacked the protein ICP27, suggesting that ICP27 inhibits GSDME-driven pyroptosis by a still-unknown mechanism. Intriguingly, a recent study showed that GSDME is required for the influx but not efflux of molecules using fluorescently labelled dextrans in a murine fibrosarcoma cell line, suggesting a previously unrecognised biology of GSDME pores [180].

#### 2.4.5. Functions of Full-Length GSDMs

While most of the known GSDM biology involves protein cleavage, liberation of the cytotoxic N-terminal domain, and subsequent cell death, a recent study suggested a function for full-length GSDMs [181]. GSDMB is abundant in inflamed mucosa of subjects with inflammatory bowel diseases. It was proposed, at least in vitro, that full-length GSDMB promotes intestinal epithelial cell proliferation and migration by boosting phosphorylation of the focal adhesion kinase FAK, via a poorly understood mechanism [181]. It is important to mention that GSDMB full-length binds phosphoinositides and sulfatide (present in the membrane of epithelial cells) with a similar binding activity to GSDMB^NT^, suggesting that the GSDMB^CT^ is not able to prevent GSDMB binding as observed for other GSDMs [164]. Similar to GSDMB, it was also reported that full-length GSDMD promotes the release of IL-1β within extracellular vesicles from intestinal epithelial cells stimulated with LPS and ATP [182]. These studies are uncovering GSDMs functions unrelated to lytic cell death and reveal new roles of these proteins.

### 2.5. Physiological Relevance of Gasdermin-D

Since 2015, when it was demonstrated that mice lacking GSDMD were protected upon LPS-endotoxic shock [109], it was clear that this pro-inflammatory protein had to be involved in many other diseases driven by inflammation. Cryopyrin-associated periodic syndromes (CAPS) are diseases strongly associated with excessive IL-1β and IL-18 production [17,183,184]. In a severe CAPS mouse model, *Gsdmd^−/−^* mice showed lesser systemic inflammation and organ damage compared to WT mice [185]. Similarly, *Gsdmd* deficiency protected the mice from autoinflammatory pathologies in a mouse model of familial Mediterranean fever, a disease caused by the mutation in the pyrin inflammasome [186]. In other chronic inflammatory diseases of the brain, *Gsdmd^−/−^* mice showed protection from experimental autoimmune encephalomyelitis and inflammatory demyelination in the central nervous system [187,188]. They also showed reduced infarct size, improved cardiac function, and increased post-survival after acute myocardial infarction compared to WT counterparts [189]. Similar to the LPS-endotoxic shock results, *Gsdmd^−/−^* mice are protected from polymicrobial sepsis induced by cecal ligation and puncture procedure [190,191]. Counterintuitively, *Gsdmd^−/−^* mice showed enhanced mortality and lupus-like clinical phenotypes compared to WT controls in an imiquimod-induced model of systemic lupus erythematosus [192]. Studies focused on colitis models have reported conflicting results related to the GSDMD contribution in the pathogenesis [182,193,194]. For instance, GSDMD plays a crucial role in limiting experimental colitis by restricting cGAS-dependent inflammation [193]. However, mice lacking GSDMD had lower colitis severity compared to WT controls [182,194]. These differences might in part be caused by the different protocols used to induce colitis, or by the different gut microbiota composition between mouse colonies and genotypes.

Several seminal studies also highlighted a non-pyroptotic functions of the GSDMD pore. Two described key GSDMD roles during disseminated intravascular coagulation after lethal endotoxin sepsis models, but they described two different mechanisms of GSDMD function [195,196]. One proposed that GSDMD promotes the release of microvesicles containing the initiator of coagulation tissue factor (TF) [195], while the second suggested that GSDMD pore formation enhances the pro-coagulant TF activity, independently of a GSDMD-driven cell death [196]. A later study also observed similar dependency on GSDMD in driving systemic coagulation and tissue injury in a mouse model of bacterial sepsis [197]. Recently, another non-pyroptotic function of GSDMD^NT^ was reported, where GSDMD pores promote mucin granule exocytosis without inducing pyroptosis in goblet cells [198]. In this model, GSDMD plays a crucial role in epithelial cell mucin secretion and mucus layer formation, required for efficient clearance of enteric pathogens. These studies, together with earlier work suggesting a role of sub-lytic GSDMD pores in IL-1β release without cell death [121], demonstrate that GSDMD pore formation at a sub-lytic level without concomitant cell death is sufficient to trigger significant physiological responses in murine models of diseases.

While largely detrimental in sterile inflammation, with some exceptions, GSDMD-induced cell death is as an important protective mechanism that regulates pathogen clearance during infection. Some examples where GSDMD death was useful, as it destroyed replicative niches for pathogens, are bacterial and viral infections with ΔsifA *S. Typhimurium*, *Brucella abortus*, *Francisella novicida* (*F. novicida*), *Y. pseudotuberculosis*, *Burkholderia cenocepacia*, *Legionella pneumophila*, *Toxoplasma gondii*, *Staphylococcus aureus*, *S. flexneri,* rotavirus and norovirus [132,167,199,200,201,202,203,204,205,206,207]. A unique function of GSDMD has been demonstrated in *F. novicida* infection, where GSDMD pores allow potassium efflux that inhibits cGAS and subsequent type-I interferon response [208].

Because the pore-forming activity of GSDMD is regulated by apoptotic caspases, i.e., it is activated by caspase-8 [149,150,151,152], but inactivated by caspases-3/7 [148,149], the physiological relevance of GSDMD is emerging in caspase-8-driven pathologies. For example, in TNF-induced lethality model in mice, where the lethal phenotype relies on caspase-8 [209,210], *Gsdmd^−/−^* mice are protected, while mice harbouring the caspases-3/7 cleavage-resistant GSDMD variant (*Gsdmd^D88A^* mice) showed poorer survival compared to their WT counterparts [211]. *Casp1^−/−^* mice showed comparable kinetics of response to WT controls, suggesting that caspase-8- and not caspase-1-driven GSDMD activation is the cause of the observed lethality in this model. The fact that TNF-induced lethality shows a GSDMD dependency, where *Gsdmd^D88A^* mice succumb with faster kinetics compared to WT counterparts, and *Gsdmd^−/−^* mice are protected [191,211], highlights GSDMD as a possible therapeutical candidate for TNF-associated diseases such as rheumatoid arthritis.

## 3. Pyroptosis in Rheumatoid Arthritis

### 3.1. Inflammation in Rheumatoid Arthritis

Rheumatoid arthritis (RA) is an inflammatory disease that affects around 1% of the population worldwide and places a large burden on health care systems, increasing morbidity and mortality in society (broader reviews about RA were published in [212,213]). Anti-inflammatory drugs, glucocorticoids and biologics such as TNF antagonists have significantly improved the quality of life of many RA patients; however, a large proportion of them (around 50%) do not respond well to current therapies, become refractory to the treatment, or present increased risk of infections. RA is characterised by progressive joint destruction and chronic inflammation of the synovium in joints. The synovium is a tissue that surrounds and encapsulates all diarthrodial (free-moving) joints and protects the intra-articular space and the synovial fluid. The synovial fluid contains hyaluronic acid and lubricin, and provides elasticity, physical lubrication, and joint support in the intra-articular space [214]. In the synovium of the healthy joint, two layers are observed: the lining layer, made of tightly packed fibroblasts and macrophages; and the sub-lining layer, made of sub-lining fibroblasts, macrophages, and adipocytes. Within the lining layer, macrophages establish epithelial-like tight junctions amongst themselves to create a physical barrier that shields the intra-articular space from immune cell infiltration [215].

During the development of RA, several key events (‘checkpoints’) have to take place, driving chronic synovial inflammation in the joints [213]. The first ‘checkpoint’ is broken with the systemic breakdown of T and B cell tolerance. In the second ‘checkpoint’, the disease transitions from asymptomatic autoimmunity to acute tissue inflammation. In the final, third ‘checkpoint’, the acute disease converts to self-sustained chronic synovitis. The last checkpoint relies on the local tissue environment, and it is associated with functional changes in tissue resident cells such as fibroblasts and macrophages. In chronic synovitis, the integrity of the synovial lining layer is destroyed and tight junctions between the lining layer macrophages are lost [215]. The compromised lining barrier allows for the infiltration of cells such as fibroblasts into the intra-articular space. Infiltrated fibroblasts become activated and upregulate invasive and tissue remodelling gene expression programmes [216]. Sub-lining fibroblasts, on the other hand, proliferate, causing synovial swelling [217], and switch to pro-inflammatory gene expression programs, resulting in the recruitment of myeloid cells such as monocytes and neutrophils from the blood. These cells first infiltrate into the synovium and then into the intra-articular space through the compromised lining layer [214,216,218,219]. The end result is a combination of synovial inflammation and intra-articular tissue damage.

Inflammasome activation and subsequent pyroptosis trigger inflammation and recruitment of myeloid cells in several chronic inflammatory diseases, so it is tempting to speculate that these inflammatory signalling pathways could also contribute to immunopathology in RA. RA is known to be driven by TNF, and it was recently shown that mice lacking the pyroptosis executor GSDMD are protected against a model of TNF-induced shock [191,211], highlighting GSDMD as a possible therapeutical candidate in RA. In the next sections, we firstly summarise published studies focused on the role of the NLRP3 inflammasome in RA. After, we discuss recent findings where the role of gasdermins has been investigated in various models of RA.

### 3.2. Role of the NLRP3 Inflammasome in Rheumatoid Arthritis

Overactive NLRP3 inflammasome has been associated with the initiation, progression and pathology of diverse chronic inflammatory, metabolic and neurodegenerative diseases (reviewed in detail in [15,220,221]). It triggers inflammation by inducing cell death and release of pro-inflammatory cytokines that recruit and activate other myeloid cells. Thus, it is of major interest to investigate whether mice lacking components of the NLRP3 inflammasome pathway may be protected in murine models of RA, especially following reports of increased NLRP3 and IL-1β levels in the synovium of patients with RA [222,223,224,225,226]. Additionally, it is known that monosodium urate (MSU) and calcium pyrophosphate dihydrate (CPPD) crystals, responsible for acute-gout arthritis and inflammation in joints, activate the NLRP3 inflammasome [227], where anti-IL-1β therapy significantly improves symptoms in patients [228,229,230,231].

The first mouse experiments of this kind were performed more than a decade ago, where two studies found unexpected protection against arthritic symptoms in *Asc^−/−^* mice, but not in *Nlrp3^−/−^* or *Casp1/11^−/−^* mice [232,233]. A first study using a collagen-induced arthritis model proposed an inflammasome-independent role for ASC in dendritic cells. In this study, ASC was somehow required for T-cell priming, auto-antibody generation and arthritic pathology [232]. A second study on mBSA-mediated antigen-induced arthritis (AIA) [233] showed that auto-antibody production is intact in *Asc^−/−^* mice, but rather that splenocytes of mBSA-immunised *Asc^−/−^* mice have decreased proliferation and secrete less pro-inflammatory IFN-γ and more anti-inflammatory IL-10 compared to WT controls. Interestingly, IL-1β blockade was protective against AIA development, but *Casp1^−/−^* deletion was not [233], suggesting that caspase-1 is not the sole protein processing pro-IL-1β in this model. A later study corroborated these early findings by showing that cartilage and bone destruction were decreased in mice lacking ASC in collagen-induced arthritis, but not in collagen-antibody-induced arthritis [234]. In the collagen-antibody-induced arthritis, the disease is induced by systemic administration of pre-formed anti-collagen autoantibodies, and hence, the immunisation step, T-cell priming and generation of endogenous autoantibodies are all bypassed [234]. Contrary to these early studies, which generally dismissed a role of NLRP3 in RA, a more recent report showed that treatment with the selective NLRP3 inhibitor MCC950 alleviates paw swelling, synovial inflammation, cartilage erosion and IL-1β levels in the same model [235]. Possible explanations for these differences in the results might rely on the mouse background, the protocol and model for arthritis induction, the timing of NLRP3 inhibition, or potential off-target effects of MCC950 in this in vivo model.

Finally, a critical role for NLRP3 inflammasome in RA was clearly described in the spontaneous polyarthritis model, which develops in mice lacking the RA susceptibility gene A20/Tnfaip3. In this model, myeloid cells lack A20, a crucial negative feedback regulator of NF-κB signalling pathway. The NF-κB pathway is responsible for transcriptional upregulation of NLRP3 and pro-IL-1β [236]. Thus, when *Nlrp3^−/−^* or *Casp1/11^−/−^* mice were crossed with A20-deficient mice, they showed protection from spontaneous erosive polyarthritis (swelling and redness of all paws) compared to WT controls [237]. Similarly, deletion of MYD88 (through which IL-1 receptor signals), but not of TNFR1, also rescued *A20*-deficient mice from arthritis-like pathology, further supporting a role of the inflammasome pathway in this model. In summary, *Nlrp3^−/−^* mice did not show protection in collagen- and mBSA-induced arthritis, while a NLRP3-specific inhibitor reduced inflammation in collagen-induced arthritis. However, NLRP3 deficiency protects *A20*-deficient mice from spontaneous polyarthritis. These results suggest that the contribution of NLRP3 inflammasome strongly depends on the murine RA models and the timing of NLRP3 inflammasome inhibition. Importantly, the role of the inflammasome component ASC during T and B cell priming needs to be considered in future studies on inflammasomes, when the RA model requires an immunisation step.

### 3.3. Role of Gasdermins in Rheumatoid Arthritis

The findings that GSDMs are pyroptotic executor proteins, and that they are activated not only by inflammasomes, but also by several other inflammatory enzymes and pathways (discussed above), made them interesting candidates to be studied in RA models. Since GSDMD deficiency protects mice upon TNF-induced shock [191,211], and RA is known to be driven by TNF [191], it is tempting to speculate that deletion of GSDMD, or other GSDMs, could be protective upon arthritis induction in vivo. Recently, several studies have been published connecting RA and GSDMs, which we summarise below.

In the serum transfer model of RA, pre-made autoantibodies are transferred from K/BxN mice into naïve mice and inflammatory arthritis is driven by myeloid cells and fibroblasts [238,239]. In this model, *Gsdmd* and *Gsdme* mRNA transcripts are enhanced in the paws of treated mice compared to WT counterparts [240], but mice lacking GSDMD show equal levels of joint swelling, bone destruction and osteolysis compared to WT controls. Mice lacking GSDME were not investigated. However, in the same study, *Gsdmd^−/−^* mice showed less articular cartilage loss and synovitis, and attenuated subchondral bone sclerosis in a model of post-traumatic arthritis, caused by meniscal ligamentous injury [240]. While these findings suggest a role of GSDMD in injury-induced rather than in antibody-induced arthritis, another preprint study employed the chemical GSDMD inhibitor disulfiram (DSF) in a type II collagen-induced arthritis model [241,242], and observed reduction of disease incidence, loss of cartilage, joint swelling and pro-inflammatory cytokines (IL-1β, IL6, IL-8 and TNF) upon DSF treatment [241]. Results were not confirmed in *Gsdmd^−/−^* mice, presumably due to the unavailability of *Gsdmd^−/−^* mice on the background (DBA/1J) required for the full penetrance of disease. A second study also used DSF to investigate the role of GSDMD in joint inflammation and found increased protein levels of GSDMD and its active form in neutrophils from peripheral blood of RA patients [241]. Serum from RA subjects could induce NETosis even in healthy bone-marrow-derived neutrophils, a process previously described to be GSDMD-dependent [132,133,134]. When the supernatant of these NETotic neutrophils was added to human fibroblasts from RA subjects, enhanced activation and proliferation of the fibroblasts were observed. These data suggest that GSDMD-driven NETosis in neutrophils and subsequent fibroblast activation could be contributors to inflammation in RA. Unfortunately, these results were not validated genetically using supernatant from *Gsdmd^−/−^* neutrophils. Off-target effects of DSF cannot be excluded, as it has been recently shown that the active metabolite of DSF also inhibits ASC speck formation in macrophages and a GSDMD-independent cell death [243]. Moreover, previous studies have shown that DSF blocks caspase activity and the NF-κB pathway [244,245,246,247,248,249], and a recent study showed profound changes in gene expression related to innate immune functions in cells treated with DSF [250].

The role of GSDME in collagen-induced arthritis has also been investigated in one study [251]. The authors reported enhanced expression of CASP3 and GSDME (full-length and N-terminal fragment) in synovial tissue macrophages from RA patients compared to samples from patients with osteoarthritis. Purified blood monocytes from RA patients also showed increased GSDME levels and underwent pyroptosis with faster kinetics compared to healthy monocytes [251]. Interestingly, prolonged stimulation with TNF sensitized blood monocytes and PMA-differentiated THP-1 cells to undergo a CASP3- and GSDME-dependent lytic cell death. Thus, authors proposed that elevated levels of TNF may promote GSDME-driven pyroptosis and inflammation in RA. To support this model, collagen-induced arthritis was induced in *Gsdme^−/−^* mice [251]. These mice developed less incidence of arthritis, decreased clinical arthritis scores, synovitis and had less pro-inflammatory synovial cytokines levels (TNF, IL-1β, IL-6). Autoantibody generation levels after immunization were equal in both mouse strains, showing that the immunisation step is not influenced by GSDME. This early study highlights a possible role of GSDME in triggering arthritic pathology in murine collagen-induced arthritis but remains to be validated by others and further mechanistically explored.

### 3.4. Targeting the NLRP3 Inflammasome and Pyroptosis in Rheumatoid Arthritis

As discussed in the previous sections, there is evidence both for and against a role of the NLRP3 inflammasome in RA, depending on the animal model used and the time of NLRP3 inhibition. In humans, multiple genetic polymorphisms of NLRP3 have been reported to correlate with increased susceptibility to RA and worse response to treatment (Table 1 in [252]). Approaches to modulate inflammasomes and pyroptosis by inhibiting NLRP3 and caspase-1 have failed in clinical trials so far, due to unfavourable toxicity (review in detail in [252,253,254]). A second strategy is to neutralize pro-inflammatory pathways triggered upon pyroptosis induction. For instance, the IL-1 receptor antagonist anakinra was tested as a therapy for RA; however, blockade of the TNF pathway was more efficient [252,254]. Blockade of IL-1β, or IL-1 receptor, had significant success in patients with CAPS, systemic juvenile idiopathic arthritis and gout. Both the blockade of IL-1β and TNF signalling pathways are associated with an increased risk of infection in patients, and a significant number of patients do not respond well to either of these therapies. Thus, novel strategies to inhibit pro-inflammatory outcomes of pyroptosis are needed.

GSDMD-driven pyroptosis contributes to inflammation in diverse in vivo models (discussed in 2.5. *Physiological relevance of Gasdermin-D*). Thus, chemical inhibition of this protein may be sufficient to damp pro-inflammatory signalling pathways, highlighting GSDMD as a possible therapeutical candidate downstream of inflammasomes and caspases. In the last few years, different molecules have been reported to inhibit GSDMD pore-forming activity by diverse mechanisms. One strategy is to develop drugs that bind key amino acids in GSDMD required for its pore-forming function. For instance, the cysteine amino acid at position 191 in human GSDMD (C192 in mouse) is crucial for its oligomerization and pore formation [116]; thus, molecules that bind to this site may modulate pyroptotic outcomes. One example is necrosulfonamide (NSA), known to block necroptosis in human cells [255]. NSA binds directly GSDMD at C191 and blocks its oligomerization and pore formation [256]. Similarly, tetraethylthiuram disulfide (disulfiram, DSF), a drug used to treat chronic alcohol dependency, was also found to inhibit pyroptosis by binding human GSDMD at C191 [242]. Furthermore, dimethyl fumarate (DMF), known to decrease inflammation and used in therapies against multiple sclerosis, triggers modifications (succination) in C191 and other cysteines of GSDMD, disrupting its pore forming activity [140]. Other molecules have also been reported to affect GSDMD pore-forming activity without affecting upstream events such as caspase-1 activation upon inflammasome assembly. For instance, in a screening for inhibitors of NETosis, LDC7559 was found to block GSDMD^NT^ pore-forming activity, thus inhibiting pyroptosis in human and murine cells [134]. The pomegranate extract punicalagin was also reported to stabilize lipids on the plasma membrane and impair GSDMD-driven pore formation [257,258]. The fact that DSF and DMF are drugs that are clinically approved and used in other therapies suggests that these drugs may be successfully used for other pyroptosis-related diseases without presenting unfavourable pharmacology and toxicity. It is key to continue characterizing them to avoid unwanted side-effects when used in other clinical settings, and to validate their specificity in different cell lines and models. We also need to consider that experiments in vitro have shown that cells lacking GSDMD undergo other form of cell death such as apoptosis, secondary necrosis or GSDME-driven pyroptosis [243,259,260], which may contribute to inflammation in vivo. However, the fact that *Gsdmd^−/−^* mice are protected in diverse inflammatory disease models in vivo, as we have seen, highlights GSDMD as a promising therapeutical candidate. The discovery of GSDMD as pyroptotic executor downstream of inflammasomes and caspases opens a new venue of possible therapies for inflammatory diseases such as RA.

### 3.5. Conclusions

In summary, in mice, GSDMD was dispensable in the K/BxN model of RA, but it showed protection in a post-traumatic osteoarthritis model, and chemical inhibition of GSDMD by DSF protects mice upon type-II collagen-induced arthritis induction. In the case of GSDME, its deficiency leads to protection against collagen-induced arthritis. It is possible that GSDME compensates for GSDMD, when GSDMD is lacking, as observed in recent studies [243,259,260]. GSDME is activated upon inflammasome activation when GSDMD is absent, leading to IL-1β release in the presence or absence of lytic cell death, depending on the endogenous GSDME expression level [259]. It is also possible that in cells lacking GSDMD, other cell death pathways could be triggered to drive lytic death and inflammation. For instance, macrophages lacking GSDMD can undergo caspase-1-driven secondary necrosis independently of GSDME [261]. More studies comparing *Gsdmd^−/−^*, *Gsdme^−/−^* and *Gsdmd^−/−^Gsdme^−/−^* mice in various RA models would be required to investigate this point. Considering that in murine models a complex cascade of pathways with different cytokines and cell types are involved in the induction of RA, it is challenging to study the contribution of a single protein in this complex system. Future studies may consider the use of immunisation-independent models of RA, to rule out any differences in immune priming between mouse strains used. These would also allow the analysis of the cellular cross-talk between synovial tissue resident cell types such as macrophages and fibroblasts in health and in chronic inflammation. In the next section, we discuss the role of fibroblasts and macrophages in driving immunopathology in RA, and how this cross-talk regulate inflammasomes and inflammation.

## 4. Role of Cell Death in Fibroblast–Macrophage Crosstalk in Rheumatoid Arthritis

### 4.1. Fibroblast and RA Inflammation

The main contributors to sustained chronic inflammatory environment in RA joints are macrophages, infiltrating monocytes and activated fibroblasts (reviewed in detail in [262,263]). In homeostatic conditions, synovial fibroblasts are an important source of the extracellular matrix components and are the producers of the synovial fluid needed to lubricate the joins and protect the cartilage surfaces. During RA, the fibroblasts phenotype changes dramatically: they become more resistant to apoptosis, increase proliferation and drive the formation of an invasive hyperplastic tissue named pannus [264,265]. Expansion of RA-associated fibroblasts correlates with the severity of the disease, and it is driven by macrophage-derived factors such as TNF, IL-1β and alarmins [266,267].

The exposure of fibroblasts to IL-1β and TNF not only promotes their proliferation but also stimulates their production of RA-exacerbating factors such as IL-6, IL-8, matrix-metalloproteases (MMPs) and reactive oxygen species [268,269,270], as well as chemokines (CCL2, CCL5, CCL8, CXCL5, CXCL10) that increase immune infiltration and activation (reviewed in detail in [265,271,272]). The production of chemokines such as CCL19 and CCL21, and the cytokine IL-7, also points towards fibroblasts as being causal in the formation of ectopic lymphoid structures in the joints. One of the main manifestations of RA that allows for even more tissue invasion by infiltrating immune cells and the expansion of fibroblasts is the production of tissue-matrix-degrading enzymes called MMPs by fibroblasts [273], in particular MMP-1, -8 and -13 [274,275]. Finally, changes in fibroblast glucose metabolism also contribute to the development of RA (reviewed in detail in [276]).

Fibroblasts are not only passive responders to pro-inflammatory cues sent by macrophages and other infiltrating immune cells, but they can also drive RA, independently of immune cells, and exacerbate disease severity when transferred to inflamed joints [216]. The persistence of the fibroblast’s aggressive phenotype may be explained by changes in their epigenetic patterns. A recent study showed that epigenetic changes in a specific subset of fibroblasts (Thy1^pos^) located in sub-lining layer are sufficient to keep a previously inflamed tissue highly ‘primed’ and susceptible to subsequent inflammation [277], explaining why inflammatory attacks tend to recur in the same joints, and highlighting the roles of specific subsets of cells in driving inflammation in RA.

### 4.2. Newly Discovered Subsets of Fibroblasts and Macrophages and Their Functions

Recent single-cell RNA-seq technologies combined with tissue imaging identified several subsets of fibroblasts with a distinct location and function in the RA synovium [216,218,278,279,280]. In general, lining layer fibroblasts can be defined in mice as FAPa^pos^PRG4^pos^Thy-1^neg^ cells, which are present in both RA and OA, and are also expanded in OA [216]. They upregulate similar functional programs in active RA and OA and are associated with bone and cartilage remodelling and production of matrix-degrading enzymes. The tissue destructive function of this subset was confirmed when lining layer fibroblasts were purified and transferred from joints of mice with active RA into the joints of naïve mice [216]. In naïve mice, transferred lining layer RA fibroblasts contributed to bone and cartilage degradation and tissue destruction, but not to synovial inflammation and swelling, suggesting a subset-specific function of fibroblasts in driving different aspects of RA pathology. The second subset of fibroblasts identified in mice are the synovial sub-lining layer fibroblasts, defined as the FAPa^pos^Thy-1^pos^ cells. They are expanded in RA, but not in OA, and contribute to synovial inflammation and swelling but not to bone and cartilage degradation and tissue destruction [216]. The inflammatory function of this subset was also confirmed in cell transfer experiments, where purified sub-lining RA fibroblast, when transferred from mice with RA to naïve mice, caused inflammation and swelling, instead of bone or cartilage destruction. A similar broad functional specialization of synovial fibroblasts was found by other groups in mice and in humans as well [218,278,279,280], even though further division of subsets and cluster names may differ (reviewed in detail in [214]). For example, not all sub-lining fibroblasts are pro-inflammatory, and some subsets, such as DKK3^pos^ sub-lining fibroblasts, are associated with pro-resolving functions and may help the repair of the damaged lining layer barrier [278]. Regardless, the general positional and functional division of fibroblast on lining and sub-lining subsets in RA synovium appear to be conserved between mice and humans and between studies.

Similarly, it was recently revealed that a phenotypic and functional heterogeneity of macrophages exists when synovial human tissues were compared from healthy controls with active RA patients (treatment naïve or treatment refractory) and patients in remission [267]. MerTK^pos^ macrophages were identified as the main subset associated with healthy synovium and disease remission, while MerTK^neg^ macrophages were the subset associated with active or treatment refractory RA. MerTK^pos^ cells are then further divided into two subsets called MerTK^pos^TREM2^pos^ and MerTK^pos^LVYE1^pos^ in humans [267]. From these, MerTK^pos^TREM2^pos^ cells correspond phenotypically to the mouse lining layer macrophages (CX3CR1^pos^), while MerTK^pos^LVYE1^pos^ cells correspond phenotypically to mouse sub-lining macrophages (RELMα+) [215]. Both lining-layer and sub-lining macrophages originate from the common local synovial self-renewing precursor (MHCII^pos^ macrophages) [215,281]. Many of the anti-inflammatory genes, including MerTK itself, that were detected in heathy human macrophages, were also found in resident lining and sub-lining macrophages in healthy mice [215], suggesting their general tissue maintenance and anti-inflammatory function. Similar subsets were described in other studies of human and mouse synovial macrophages. Some examples are another single-cell RNA-seq analysis of human RA synovium that identified MerTK^pos^ cells as an M2 cluster [278], the analysis of total human synovial CD14^pos^ cells exposed to anti-inflammatory medications used to treat RA (anti-TNF, tofacitinib, and dexamethasone) that upregulated MerTK [282], or the analysis of mouse synovial macrophages associated with disease resolution in a model of serum transfer-induced RA [219]. Thus, resident macrophages found in healthy synovium are well-conserved between mouse and humans and between studies and can be broadly identified as MerTK^pos^ cells, with further division to lining and sub-lining subsets in both human and mice.

MerTK^neg^ inflammatory macrophages in humans are associated with active and treatment-refractory RA [267]. These cells correspond phenotypically to mouse inflammatory subsets that infiltrate the synovium from the blood during active disease. Infiltrating myeloid cells are identified in mice as monocytes (Ly6c^pos^) and inflammatory macrophages (CCR2^pos^) [215]. They have a pro-inflammatory profile and high expression of inflammasome components and pro-IL-1β. Similar gene programs were also found in other studies of human RA macrophages, which identified IL-1β^pos^ cells as an M1 cluster [278]. Thus, synovial macrophages associated with active diseases are also well conserved between mouse and humans, as well as between studies, and can be broadly identified as a MerTK^neg^ IL-1β^pos^ subset in humans, and CCR2^pos^IL-1β^pos^ infiltrating macrophages in mice.

Overall, all these studies have revealed inflammatory and anti-inflammatory functions of distinct macrophages and fibroblasts subsets. The exact mechanisms by how these cell subsets regulate inflammation in RA, and whether one cellular subset can regulate the inflammatory response of another subset, is a topic of high interest, which is further discussed below.

### 4.3. Fibroblast–Macrophage Interactions in Synovium

The cross-talk between fibroblasts and macrophages regulates homeostasis and normal organ development [283,284], but when it is dysregulated, it drives tissue damage and pathologic inflammation in many chronic inflammatory diseases (reviewed in detail in [271]). The development of approaches to collect and analyse synovial cells has allowed researchers to explore the gene expression profiles of fibroblast and macrophages found in the synovium of healthy people and compare them to those found in active and treatment refractory RA, or in disease remission tissues (reviewed in detail in [214,285,286]. The types of cell–cell interactions that maintain a healthy synovium and those that drive disease are beginning to emerge.

Macrophages and fibroblasts generally have anti-inflammatory and tissue-remodelling gene expression profiles in the healthy lining layer [215], but what signals they exchange to maintain these profiles is poorly understood. The sub-lining layer of the synovium is further away from the intra-articular space and is in contact with blood and lymphatic vessels. This layer is thought to be the port of entry for nutrients, but also for immune cells during RA flares. Precise positioning of fibroblasts and macrophages within the two layers of synovium matters (discussed in detail in [287]); it dictates the type of interactions they establish, the tissue microenvironment they sense and respond to, and the type of cell fates and functional programmes they develop [279,281]. One example of how functional identity is imprinted by tissue location in synovial cells is the sub-lining fibroblasts that are located in the close proximity to endothelial cells and blood vessels [279]. Endothelial cells are the source of Notch ligands that dictate the phenotypic identity of nearby Thy1^pos^ sub-lining fibroblast. Only cells further away from the Notch gradient are able to take on the distinct functional identity characteristic of the lining layer Thy1^neg^ fibroblasts. Resident macrophages populate the synovial tissue during embryonic development, and they self-renew locally to maintain the pool during life [215]. Resident fibroblasts also self-renew and likely differentiate from a precursor common to all tissue types including the joint [288].

**Figure 2:**
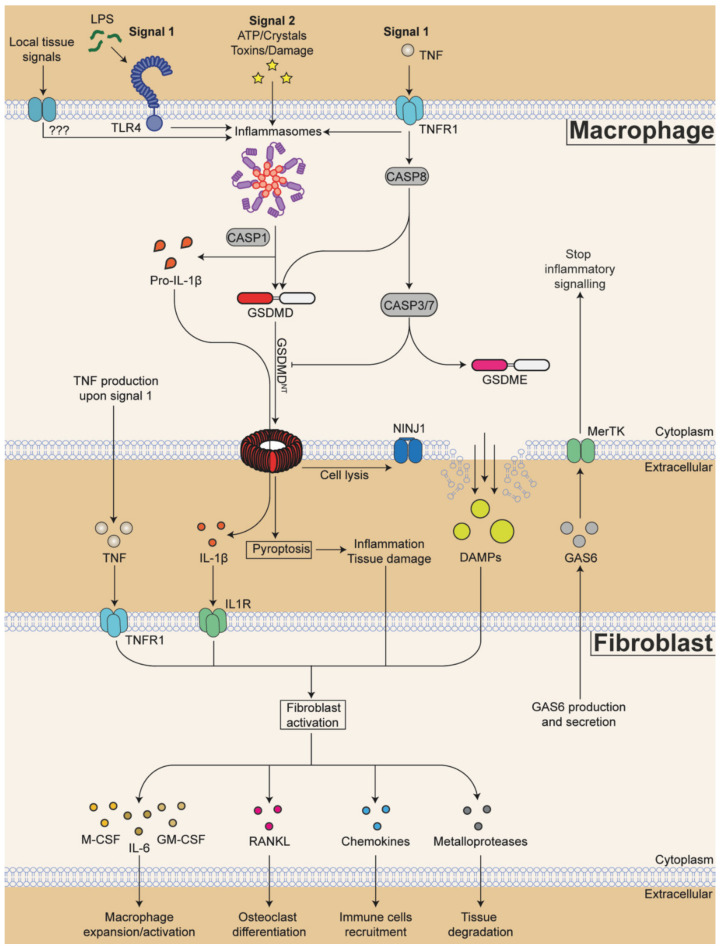
Proposed model of macrophage-fibroblast cross-talk during rheumatoid arthritis. The priming of NLRP3 expression starts with the detection of pathogen- and damage-associated molecular patterns (DAMPs) such as lipopolysaccharide (LPS) and tumour necrosis factor (TNF) (signal 1). Other local tissue signals may be sensed by other receptors that might prime other inflammasomes during rheumatoid arthritis. Inflammasome activation occurs when cells are exposed to a second signal (signal 2) such as high extracellular levels of adenosine triphosphate (ATP) released during tissue damage, bacterial toxins, pathologically accumulated insoluble crystals or other tissue damage signals. Active NLRP3 inflammasome is composed of the sensor NLRP3 (purple), the adapter protein ASC (red) and the effector enzyme caspase-1 (CASP1). Active CASP1 cleaves pro-inflammatory cytokines pro-interleukin (IL)-1β and pro-IL-18 into their biologically active forms. CASP1 also cleaves the pore-forming protein gasdermin-D (GSDMD), to liberate its N-terminal domain (GSDMD^NT^, in red) from its autoinhibition by the C-terminal domain (in white). Once liberated, GSDMD^NT^ forms pores at the plasma membrane that allow the release of cleaved cytokines and also drive inflammatory cell death pyroptosis. Nerve injury-induced protein 1 (NINJ1) is required for final cell lysis and release of bigger DAMPs. Caspase-8 (CASP8), activated downstream of TNF receptor (TNFR1), can cleave GSDMD as the same site as CASP1 to drive pyroptosis. On the other hand, CASP8 also activates caspases-3/7 (CASP3/7) that can inhibit GSDMD^NT^ by cleaving within its N-terminal pore-forming domain. In cells with high GSDME expression, CASP3/7 can cleave and activate gasdermin-E (GSDME), another pore forming protein of the gasdermin family. TNF, IL-1β and other DAMPs are sensed by fibroblasts, which express corresponding receptors. Activated fibroblasts in turn secrete cytokines, chemokines and metalloproteases that promote further synovial inflammation. For example, fibroblast-derived macrophage colony-stimulating factor (M-CSF), granulocyte-M-CSF (GM-CSF), and IL-6 promote macrophage expansion and activation. Receptor activator of nuclear factor kappa-Β ligand (RANKL) promotes osteoclast differentiation and bone resorption. Chemokines CC-chemokine ligand (CCL)2, CCL5, CCL8, CXC-chemokine ligand (CXCL)5 and CXCL10 increase immune cells recruitment into the inflamed joints. Metalloproteases induce tissue matrix degradation. On the other hand, fibroblasts can produce and secrete growth arrest-specific protein 6 (GAS6) that acts on the TAM receptor MerTK on macrophages to terminate further inflammatory signalling. How and when these factors that coordinate disease magnitude and duration are released and exchanged between fibroblasts and macrophages is still poorly understood.

Analysis of gene expression in human RA synovium [278], and analysis of other tissues, such as the gut [289], has allowed us to hypothesize a model of macrophage-fibroblast crosstalk in RA (Figure 2), where the highest source of IL-1β are likely infiltrating monocyte-derived macrophages and neutrophils. They express the highest levels of all components of inflammasome signalling machinery and of pro-IL-1β, and they are also the biggest producers of TNF in RA [290]. Macrophage-derived TNF and IL-1β then activate fibroblasts (Figure 2), which express the highest levels of IL-1R in RA synovium, according to the gene expression data [278]. Activated fibroblasts in turn proliferate and initiate tissue-destructive and inflammatory programmes and secrete chemokines such as CCL2 to recruit more monocytes. They also secrete macrophage colony-stimulating factor (M-CSF) to support macrophage expansion, and IL-6 and granulocyte-M-CSF (GM-CSF) to promote further macrophage activation (Figure 2) (reviewed in detail in [291]). Finally, fibroblast secrete the receptor activator of nuclear factor kappa-Β ligand (RANKL) to promote differentiation of osteoclasts, myeloid cells specialised in bone resorption. Together, activated macrophages and fibroblasts have the potential to create a positive feedback loop that sustains chronic inflammation and tissue destruction in RA (Figure 2).

A new conceptual framework allows us, in this review, to discuss, or rather hypothesize, how the cellular cross-talk between fibroblasts and macrophages may promote disease progression in RA and inflammation. We specifically focus on how this cross-talk is shaped by the signals that induce cell death (e.g., TNF), signals that sense cell death and damage (e.g., TAM receptors, inflammasomes), or signals that are produced as a result of cell death pathway activity (e.g., IL-1).

### 4.4. Cell Death Pathways in Fibroblasts

While fibroblasts can be activated by macrophages from the inflammatory microenvironment of the joints, they are also capable of initiating inflammation themselves in response to PAMPs and DAMPs. Fibroblasts express several PRRs, such as TLR2, TLR3, TLR4 and TLR5, albeit likely at a lower level than macrophages [263,292,293]. TLR3 and TLR4 are upregulated in early RA, and primary human fibroblasts can respond to LPS and Poly(I:C) by producing IL-6 and MMPs [294]. TLR4 in fibroblasts can also be activated by endogenous TLR activators such as citrullinated endogenous peptides or the extracellular matrix protein Tenascin-C [295], and TLR4 inhibition reduces fibroblast-induced cytokine production [295]. Considering the ability of fibroblasts to respond to PAMPs and their high expression of IL1R [278], it becomes interesting to know if they could themselves secrete IL-1β through inflammasome activation, thus creating an autocrine activation feedback loop. In scRNA-seq analysis of human RA synovium, no expression of *Nlrp3* or *Il1b* was found in fibroblasts, albeit other inflammasome components such as *Pycard* (ASC-encoding gene) and *Aim2* were present [278]. These results are in line with other studies that did not find *Nlrp3* upregulation in fibroblasts from RA joints [225]. On the other hand, a recent study showed that after repeated arthritic flares caused in mice by RA inducers such as MSU crystals or zymosan, *Nlrp3* was one of the most upregulated genes in fibroblasts [277]. These cells also displayed increased caspase-1 activity and IL-1β release in the supernatant, pointing to possible inflammasome activation in epigenetically reprogrammed fibroblasts in chronic disease.

While it is still unclear whether synovial fibroblasts can activate the inflammasome complex, the role of fibroblast-derived IL-1β is well established in other diseases. In the case of ischemia-reperfusion injury as an example, mouse cardiac fibroblasts upregulate *Nlrp3* and trigger the release of IL-1β and IL-18 in vitro [296,297], and *Nlrp3*-deficient mice have reduced hypoxic damage [297]. Similarly, NLRP3-driven inflammasome activation is documented in human dental pulp fibroblasts and gingival fibroblasts, where it is regulated by oral bacteria [298,299,300]. A novel role for NLRP3 inflammasome was also found in cancer-associated fibroblast where, somewhat counterintuitively, it facilitates tumour progression by increasing cell adhesion and the recruitment of suppressor cells [301]. It is then possible that, like fibroblasts in other tissues, synovial fibroblasts could begin to express and activate the inflammasome in chronic RA and thus further contribute to disease severity.

Another key feature of synovial fibroblasts is their resistance to apoptosis that, by hindering cell clearance, contribute to fibroblasts hyperplasia and invasiveness (reviewed in detail in [264]). Several metabolic and transcriptional causes for this resistance have been found. One of the main causes of resistance to apoptosis seems to be increased autophagy that, coupled with the reduced expression of DNA damage-inducible transcript 3 protein (CHOP), impairs apoptosis by limiting the ER stress response [302,303]. One of the main RA cytokines, TNF, that is normally associated with cell death, in the case of fibroblasts helps the cells escape apoptosis, via activation of the pro-survival signalling pathway NF-κB, and the upregulation of soluble Fas among other pathways [304,305,306,307,308]. In RA, soluble Fas binds to Fas ligands, thereby blocking its interaction with membrane-bound Fas receptor and preventing cell death. TNF can also push fibroblasts from apoptosis to necroptosis or necrosis in some cases [269,306,309,310,311]. These results have not always been experimentally confirmed [312,313], possibly because of the variety of effects that TNF has in different fibroblast cell lines normally used in in vitro studies [308], as well as due to differences between experimental cellular stimulation conditions [307].

Whether fibroblasts may contribute to inflammation by inducing GSDMs-mediated pyroptosis is still unknown. As synovial fibroblasts appear to express several GSDMs (based on gene expression data in [278]), it is possible that they would undergo pyroptosis during RA, either following the expression and activation of the NLRP3 inflammasome, as reported after repeated flares [277], or upon cleavage of GSDMs by an NLRP3-independent pathways, for example, downstream of the TNF-CASP8 signalling axis or upon cleavage by other enzymes such as granzymes, if delivered by activated T cells or NK cells. Pyroptotic cell death has been documented in cardiac fibroblasts following cardiac disease, in cancer-associated fibroblasts and in gingival fibroblasts [301,314,315], and most recently in joint fibroblasts as well [316]. In all these contexts fibroblast pyroptosis contributed to exacerbate inflammation.

Finally, to stop inflammation and cartilage destruction, fibroblasts could help with disease resolution. The existence of an anti-inflammatory feedback loop made of fibroblast-derived growth arrest-specific protein 6 (GAS6) acting on the macrophage TAM receptor MerTK was recently discovered, showing the potential to stop inflammation and allow disease remission [267]. In the next section, we discuss one possible mechanism by which fibroblasts may limit macrophage-driven inflammation. We mainly focus on the GAS6-MerTK signalling pathway and discuss how this signalling pathway may limit inflammation by modulating the NLRP3 inflammasome and GSDMs-driven pyroptosis.

## 5. Regulation of Inflammation via TAM Receptors in Rheumatoid Arthritis

### 5.1. TAM Receptors in Inflammation

The tyrosine kinase receptors Tyro3, Axl and MerTK belong to the TAM receptors family (reviewed in detail in [317]). These cell-surface transmembrane receptors, known to modulate inflammation and efferocytosis, share a similar domain structure containing an Ig domain that allows the binding of TAM ligands GAS6 and protein S (PROS1). Binding of these ligands to TAM receptors (TAMs) leads to TAM receptor phosphorylation and activation [318,319,320,321,322,323,324]. The affinity of ligands to each TAM receptor differs. For instance, GAS6 preferentially binds Axl and Tyro3, but has decreased affinity to MerTK, while PROS1 triggers Tyro3 and MerTK activation, but not Axl [322,323,325,326]. Diverse studies have revealed key roles for TAM receptors in limiting inflammation by diverse mechanisms. For example, TAMs activation triggers the induction of suppressor of cytokines signalling proteins 1 and 3 [327]. Another mechanism has been observed in peritoneal macrophages where GAS6 and PROS1 synergistically limit basal- and LPS-induced levels of pro-inflammatory cytokine production [328]. When TLR signalling is engaged, these cells transiently downregulate the expression of GAS6 and PROS1 to allow a proper inflammatory response, and complete neutralization of GAS6 or PROS1 results in increased levels of IL-1β, TNF and IL6 transcripts upon TLR-activation. Similarly, GAS6-driven inhibition of LPS-induced TNF and IL-6 secretion was also observed in the PMA-differentiated U937 monocyte cell line [329]. Finally, in LPS-induced endotoxic shock, *Mertk^−/−^* mice showed elevated levels of TNF in the serum and enhanced lethality kinetics compared to WT controls [330].

Since the discovery of the striking role of TAMs in limiting inflammation, the physiological relevance of this inhibitory axis was extensively studied by many diseases. Already back in 2001, a *Tyro3^−/−^Axl^−/−^Mertk^−/−^* mouse strain was generated [331]. These mice did not show any abnormalities in the immune system in the first 4 weeks after birth but developed chronic inflammation and autoimmune phenotypes as adults. Later studies have associated TAM deficiency with worse outcomes in several inflammatory diseases such as multiple sclerosis, systemic lupus erythematosus and inflammatory bowel diseases [332]. Surprisingly, a recent study showed increased protection of *Axl^−/−^* mice in a heart reperfusion infarct model [333]. The authors found that Axl promotes the switch to glycolytic metabolism and subsequent pro-IL-1β production in LPS-primed cells. In contrast to Axl, in this same model, *Mertk^−/−^* mice showed worsened cardiac repair and contractile function, which was also observed in elsewhere [334,335]. Thus, despite multiple studies showing the anti-inflammatory role of TAMs, there are examples of their pro-inflammatory roles in specific contexts. These highlight a need to keep an open mind and understand how and in which context TAMs regulate local tissue inflammation.

### 5.2. Regulation of Pyroptosis via TAM Receptors

Emerging studies have shown different contexts in which TAMs may limit the NLRP3 inflammasome and cell death. For instance, *Axl^−/−^* mice showed worse disease phenotypes in murine models of induced hepatic inflammation, which were restored to WT control levels when NLRP3 was chemically inhibited [336]. Similarly, GAS6-driven MerTK activation leads to enhanced autophagy levels, and reduced NLRP3 inflammasome assembly, in microglial cells from mice treated with an endovascular perforation to induce a subarachnoid hemorrhage [337]. Highlighting a possibility of pro-inflammatory function of TAMs in some contexts, TAMs can also promote necroptosis by inducing phosphorylation of the necroptotic executor protein MLKL [338]. Thus, mice lacking TAMs were protected from lethality induced by systemic inflammatory response syndrome, a well-known model that is driven by the necroptotic proteins MLKL and RIPK3 [210,339].

In arthritis, TAMs appear to have a general anti-inflammatory role. The TAM ligand GAS6 is produced by fibroblasts to promote anti-inflammatory responses in MerTK-expressing macrophages (Figure 2) [267]. It was shown that GAS6, secreted by sub-lining fibroblasts, acts as a ligand for MerTK on MerTK^pos^ macrophages and limits inflammation in healthy joints and in joints of patients in remission. This protective MerTK^pos^ subset (or at least the expression of MerTK receptor) is lost in patients with active and treatment-refractory RA. Furthermore, enzymes associated with active RA, such as ADAM17 (ADAM metallopeptidase domain 17), are known to cleave MerTK from the cell surface, and to promote tissue inflammation by limiting production of pro-resolving mediators in other diseases such as ischemia-reperfusion injury [340]. Since the pathology in RA is driven by pro-inflammatory cytokines such as TNF, IL-1β and IL-6, and TAM receptors regulate the production of these cytokines, it is tempting to speculate that mice lacking TAM receptors would show exacerbated arthritic pathology. In fact, TAM receptor stimulation by GAS6 and PROS1 lead to reduced pro-inflammatory cytokine production, inflammation and knee cartilage/bone erosion in mice challenged with collagen-induced arthritis [341]. Similarly, mice lacking MerTK had enhanced joint inflammation compared to WT mice in the K/BxN serum transfer arthritis model [342]. When PROS1 was overexpressed to induce TAM activation, LPS- and P3C- induced gene expression of pro-inflammatory cytokines was reduced in cells and in mouse knees.

Overall, these studies highlight the potent effect of TAMs in limiting inflammation via inhibition of pro-inflammatory cytokines in RA. Interestingly, many of these cytokines are controlled via inflammasome activation, or released upon GSDMs-driven pyroptosis. Whether and how TAMs may mechanistically limit NLRP3 activation is currently unclear and unexplored. One possible mechanism might rely on TAM-regulated induction of autophagy. It is reported that GAS6-binding to the TAM receptor Axl promotes autophagy [336], and NLRP3 inflammasome activation is known to be limited via the autophagy pathway by degrading the NLRP3 inflammasome complex (reviewed in detail in [343]). In agreement with this prediction, NLRP3 inflammasome activation was reduced when LPS-primed cells were co-stimulated with recombinant GAS6 and ATP, an effect which was dependent on the autophagy protein E1 ligase Atg7, but independent on type-I IFNs [336]. Thus, normal GAS6 production by fibroblasts may inhibit the induction of inflammatory pathways such as TLR and inflammasome signalling in RA macrophages. While the role of GAS6/MerTK axis in the fibroblast–macrophage crosstalk remains incompletely explored in RA, it is well established in fibrosis and in the tumour microenvironment, where cancer-associated fibroblasts produce abundant amounts of GAS6 with anti-inflammatory effects that promote cancer survival and migration (reviewed in detail in [344])

Finally, TAMs may limit inflammation triggered by pyroptosis by promoting the efferocytosis of dying cells. It is well established that TAMs promote efferocytosis by binding to phosphatidylserine (PS) exposed on apoptotic cells (reviewed in detail in [345]). Interestingly, PS exposure also occurs on pyroptotic and necroptotic cells [346,347]. Whether TAMs limit inflammation by promoting efferocytosis of pyroptotic cells, and what would be the functional outcome of such uptake, is unknown.

## 6. Conclusions and Perspectives

Arthritis is a complex disease with different cytokines and cell types involved. It starts with a break in B and T cell tolerance, the generation of autoantibodies, and finally, it presents as chronic joint inflammation and destruction. The last step is maintained by the cross-talk between fibroblasts and macrophages in inflamed joints. Because of such a complex disease ethology, it is challenging to study and interpret the contribution of a single protein in this complex system. Despite strong indications that components of the inflammasome machinery are upregulated and activated in inflamed human joints, mapping how exactly they contribute to local tissue inflammation and destruction has been challenging, where mechanisms and conclusions varied depending on the experimental models used. To understand the local tissue biology of inflammasomes in the joint, future studies may consider the use of immunisation-independent models of RA, such as serum- or autoantibody-transfer model. These bypass the immunisation step and eliminate any differences in T and B cell immune priming between various mouse strains used. These models also focus the analysis to the local tissue environment and the cross-talk between synovial tissue resident cell types such as macrophages and fibroblasts, which are currently poorly understood and hence difficult to target in chronic inflammation.

The emerging knowledge of GSDMs biology and regulation, highlights that we should also think beyond inflammasomes when we study pyroptosis in arthritis. Pyroptosis is an inflammatory form of cell death, and while such lytic form of cell death may be useful in antimicrobial defence, it is typically associated with pathology in sterile inflammatory diseases. Here, we discussed how various GSMDs can be activated not only by inflammasomes but also by several other inflammatory enzymes and pathways, many of which are already linked with RA. One example is finding that GSDMs can be cleaved downstream of TNF-Caspase-8 signalling and that GSDMD deficiency protects mice upon TNF-induced shock [191,211]. RA is known to be driven by TNF [191], and it is tempting to speculate how the blockade of one or more GSDMs could be protective upon arthritis induction. Beyond TNF, there are other local tissue signals in damaged joints, often highly infiltrated by neutrophils, that can activate Caspase-8 and other GSDM-cleaving enzymes. Thus, inflammatory cell death may be an important contributor to local tissue pathology in both TNF-driven and TNF-independent forms of tissue inflammation. Understanding how such unscheduled death is prevented in heathy tissues is key to knowing how to control it in disease.

## Data Availability

Not applicable.

## References

[1] Zychlinsky A., Prevost M.C., Sansonetti P.J. (1992). Shigella flexneri induces apoptosis in infected macrophages. Nature.

[2] Chen L.M., Kaniga K., Galan J.E. (1996). *Salmonella* spp. are cytotoxic for cultured macrophages. Mol. Microbiol..

[3] Monack D.M., Raupach B., Hromockyj A.E., Falkow S. (1996). *Salmonella typhimurium* invasion induces apoptosis in infected macrophages. Proc. Natl. Acad. Sci. USA.

[4] Hersh D., Monack D.M., Smith M.R., Ghori N., Falkow S., Zychlinsky A. (1999). The Salmonella invasin SipB induces macrophage apoptosis by binding to caspase-1. Proc. Natl. Acad. Sci. USA.

[5] Hilbi H., Moss J.E., Hersh D., Chen Y., Arondel J., Banerjee S., Flavell R.A., Yuan J., Sansonetti P.J., Zychlinsky A. (1998). Shigella-induced apoptosis is dependent on caspase-1 which binds to IpaB. J. Biol. Chem..

[6] Hilbi H., Chen Y., Thirumalai K., Zychlinsky A. (1997). The interleukin 1beta-converting enzyme, caspase 1, is activated during Shigella flexneri-induced apoptosis in human monocyte-derived macrophages. Infect. Immun..

[7] Brennan M.A., Cookson B.T. (2000). Salmonella induces macrophage death by caspase-1-dependent necrosis. Mol. Microbiol..

[8] Chen Y., Smith M.R., Thirumalai K., Zychlinsky A. (1996). A bacterial invasin induces macrophage apoptosis by binding directly to ICE. EMBO J..

[9] Cookson B.T., Brennan M.A. (2001). Pro-inflammatory programmed cell death. Trends Microbiol..

[10] Martinon F., Burns K., Tschopp J. (2002). The inflammasome: A molecular platform triggering activation of inflammatory caspases and processing of proIL-beta. Mol. Cell.

[11] Srinivasula S.M., Poyet J.L., Razmara M., Datta P., Zhang Z., Alnemri E.S. (2002). The PYRIN-CARD protein ASC is an activating adaptor for caspase-1. J. Biol. Chem..

[12] Stehlik C., Lee S.H., Dorfleutner A., Stassinopoulos A., Sagara J., Reed J.C. (2003). Apoptosis-associated speck-like protein containing a caspase recruitment domain is a regulator of procaspase-1 activation. J. Immunol..

[13] Pandey A., Shen C., Feng S., Man S.M. (2021). Cell biology of inflammasome activation. Trends Cell Biol..

[14] Zheng D., Liwinski T., Elinav E. (2020). Inflammasome activation and regulation: Toward a better understanding of complex mechanisms. Cell Discov..

[15] Chauhan D., Vande Walle L., Lamkanfi M. (2020). Therapeutic modulation of inflammasome pathways. Immunol. Rev..

[16] Swanson K.V., Deng M., Ting J.P. (2019). The NLRP3 inflammasome: Molecular activation and regulation to therapeutics. Nat. Rev. Immunol..

[17] Agostini L., Martinon F., Burns K., McDermott M.F., Hawkins P.N., Tschopp J. (2004). NALP3 forms an IL-1beta-processing inflammasome with increased activity in Muckle-Wells autoinflammatory disorder. Immunity.

[18] Bauernfeind F.G., Horvath G., Stutz A., Alnemri E.S., MacDonald K., Speert D., Fernandes-Alnemri T., Wu J., Monks B.G., Fitzgerald K.A. (2009). Cutting edge: NF-kappaB activating pattern recognition and cytokine receptors license NLRP3 inflammasome activation by regulating NLRP3 expression. J. Immunol..

[19] Franchi L., Eigenbrod T., Nunez G. (2009). Cutting edge: TNF-alpha mediates sensitization to ATP and silica via the NLRP3 inflammasome in the absence of microbial stimulation. J. Immunol..

[20] O’Connor W., Harton J.A., Zhu X., Linhoff M.W., Ting J.P. (2003). Cutting edge: CIAS1/cryopyrin/PYPAF1/NALP3/CATERPILLER 1.1 is an inducible inflammatory mediator with NF-kappa B suppressive properties. J. Immunol..

[21] Bezbradica J.S., Coll R.C., Schroder K. (2017). Sterile signals generate weaker and delayed macrophage NLRP3 inflammasome responses relative to microbial signals. Cell Mol. Immunol..

[22] Allam R., Lawlor K.E., Yu E.C., Mildenhall A.L., Moujalled D.M., Lewis R.S., Ke F., Mason K.D., White M.J., Stacey K.J. (2014). Mitochondrial apoptosis is dispensable for NLRP3 inflammasome activation but non-apoptotic caspase-8 is required for inflammasome priming. EMBO Rep..

[23] Gurung P., Anand P.K., Malireddi R.K., Vande Walle L., Van Opdenbosch N., Dillon C.P., Weinlich R., Green D.R., Lamkanfi M., Kanneganti T.D. (2014). FADD and caspase-8 mediate priming and activation of the canonical and noncanonical Nlrp3 inflammasomes. J. Immunol..

[24] Man S.M., Tourlomousis P., Hopkins L., Monie T.P., Fitzgerald K.A., Bryant C.E. (2013). Salmonella infection induces recruitment of Caspase-8 to the inflammasome to modulate IL-1beta production. J. Immunol..

[25] Philip N.H., DeLaney A., Peterson L.W., Santos-Marrero M., Grier J.T., Sun Y., Wynosky-Dolfi M.A., Zwack E.E., Hu B., Olsen T.M. (2016). Activity of Uncleaved Caspase-8 Controls Anti-bacterial Immune Defense and TLR-Induced Cytokine Production Independent of Cell Death. PLoS Pathog..

[26] Weng D., Marty-Roix R., Ganesan S., Proulx M.K., Vladimer G.I., Kaiser W.J., Mocarski E.S., Pouliot K., Chan F.K., Kelliher M.A. (2014). Caspase-8 and RIP kinases regulate bacteria-induced innate immune responses and cell death. Proc. Natl. Acad. Sci. USA.

[27] DeLaney A.A., Berry C.T., Christian D.A., Hart A., Bjanes E., Wynosky-Dolfi M.A., Li X., Tummers B., Udalova I.A., Chen Y.H. (2019). Caspase-8 promotes c-Rel-dependent inflammatory cytokine expression and resistance against Toxoplasma gondii. Proc. Natl. Acad. Sci. USA.

[28] Gitlin A.D., Heger K., Schubert A.F., Reja R., Yan D., Pham V.C., Suto E., Zhang J., Kwon Y.C., Freund E.C. (2020). Integration of innate immune signalling by caspase-8 cleavage of N4BP1. Nature.

[29] McKee C.M., Coll R.C. (2020). NLRP3 inflammasome priming: A riddle wrapped in a mystery inside an enigma. J. Leukoc. Biol..

[30] McKee C.M., Fischer F.A., Bezbradica J.S., Coll R.C. (2021). PHOrming the inflammasome: Phosphorylation is a critical switch in inflammasome signalling. Biochem. Soc. Trans..

[31] Perregaux D., Gabel C.A. (1994). Interleukin-1 beta maturation and release in response to ATP and nigericin. Evidence that potassium depletion mediated by these agents is a necessary and common feature of their activity. J. Biol. Chem..

[32] Surprenant A., Rassendren F., Kawashima E., North R.A., Buell G. (1996). The cytolytic P2Z receptor for extracellular ATP identified as a P2X receptor (P2X7). Science.

[33] Walev I., Reske K., Palmer M., Valeva A., Bhakdi S. (1995). Potassium-inhibited processing of IL-1 beta in human monocytes. EMBO J..

[34] Munoz-Planillo R., Kuffa P., Martinez-Colon G., Smith B.L., Rajendiran T.M., Nunez G. (2013). K^+^ efflux is the common trigger of NLRP3 inflammasome activation by bacterial toxins and particulate matter. Immunity.

[35] Petrilli V., Papin S., Dostert C., Mayor A., Martinon F., Tschopp J. (2007). Activation of the NALP3 inflammasome is triggered by low intracellular potassium concentration. Cell Death Differ..

[36] Tapia-Abellan A., Angosto-Bazarra D., Alarcon-Vila C., Banos M.C., Hafner-Bratkovic I., Oliva B., Pelegrin P. (2021). Sensing low intracellular potassium by NLRP3 results in a stable open structure that promotes inflammasome activation. Sci. Adv..

[37] Gross C.J., Mishra R., Schneider K.S., Medard G., Wettmarshausen J., Dittlein D.C., Shi H., Gorka O., Koenig P.A., Fromm S. (2016). K^+^ Efflux-Independent NLRP3 Inflammasome Activation by Small Molecules Targeting Mitochondria. Immunity.

[38] Wolf A.J., Reyes C.N., Liang W., Becker C., Shimada K., Wheeler M.L., Cho H.C., Popescu N.I., Coggeshall K.M., Arditi M. (2016). Hexokinase Is an Innate Immune Receptor for the Detection of Bacterial Peptidoglycan. Cell.

[39] Gaidt M.M., Ebert T.S., Chauhan D., Schmidt T., Schmid-Burgk J.L., Rapino F., Robertson A.A., Cooper M.A., Graf T., Hornung V. (2016). Human Monocytes Engage an Alternative Inflammasome Pathway. Immunity.

[40] Daniels M.J., Rivers-Auty J., Schilling T., Spencer N.G., Watremez W., Fasolino V., Booth S.J., White C.S., Baldwin A.G., Freeman S. (2016). Fenamate NSAIDs inhibit the NLRP3 inflammasome and protect against Alzheimer’s disease in rodent models. Nat. Commun..

[41] Domingo-Fernandez R., Coll R.C., Kearney J., Breit S., O’Neill L.A.J. (2017). The intracellular chloride channel proteins CLIC1 and CLIC4 induce IL-1beta transcription and activate the NLRP3 inflammasome. J. Biol. Chem..

[42] Tang T., Lang X., Xu C., Wang X., Gong T., Yang Y., Cui J., Bai L., Wang J., Jiang W. (2017). CLICs-dependent chloride efflux is an essential and proximal upstream event for NLRP3 inflammasome activation. Nat. Commun..

[43] Green J.P., Yu S., Martin-Sanchez F., Pelegrin P., Lopez-Castejon G., Lawrence C.B., Brough D. (2018). Chloride regulates dynamic NLRP3-dependent ASC oligomerization and inflammasome priming. Proc. Natl. Acad. Sci. USA.

[44] Chen J., Chen Z.J. (2018). PtdIns4P on dispersed trans-Golgi network mediates NLRP3 inflammasome activation. Nature.

[45] Magupalli V.G., Negro R., Tian Y., Hauenstein A.V., Di Caprio G., Skillern W., Deng Q., Orning P., Alam H.B., Maliga Z. (2020). HDAC6 mediates an aggresome-like mechanism for NLRP3 and pyrin inflammasome activation. Science.

[46] Lee B., Hoyle C., Green J.P., Wellens R., Martin-Sanchez F., Williams D., Seoane P.I., Bennett H., Adamson A., Lopez-Castejon G. (2021). NLRP3 activation in response to disrupted endocytic traffic. bioRxiv.

[47] Seoane P.I., Lee B., Hoyle C., Yu S., Lopez-Castejon G., Lowe M., Brough D. (2020). The NLRP3-inflammasome as a sensor of organelle dysfunction. J. Cell Biol..

[48] Andreeva L., David L., Rawson S., Shen C., Pasricha T., Pelegrin P., Wu H. (2021). NLRP3 cages revealed by full-length mouse NLRP3 structure control pathway activation. Cell.

[49] Hochheiser I.V., Pilsl M., Hagelueken G., Moecking J., Marleaux M., Brinkschulte R., Latz E., Engel C., Geyer M. (2022). Structure of the NLRP3 decamer bound to the cytokine release inhibitor CRID3. Nature.

[50] Matikainen S., Nyman T.A., Cypryk W. (2020). Function and Regulation of Noncanonical Caspase-4/5/11 Inflammasome. J. Immunol..

[51] Wright S.S., Vasudevan S.O., Rathinam V.A. (2022). Mechanisms and Consequences of Noncanonical Inflammasome-Mediated Pyroptosis. J. Mol. Biol..

[52] Wang B., Tian Y., Yin Q. (2019). AIM2 Inflammasome Assembly and Signaling. Adv. Exp. Med. Biol..

[53] Sharma M., de Alba E. (2021). Structure, Activation and Regulation of NLRP3 and AIM2 Inflammasomes. Int. J. Mol. Sci..

[54] Kumari P., Russo A.J., Shivcharan S., Rathinam V.A. (2020). AIM2 in health and disease: Inflammasome and beyond. Immunol. Rev..

[55] Schnappauf O., Chae J.J., Kastner D.L., Aksentijevich I. (2019). The Pyrin Inflammasome in Health and Disease. Front. Immunol..

[56] Heilig R., Broz P. (2018). Function and mechanism of the pyrin inflammasome. Eur. J. Immunol..

[57] Zhong F.L., Mamai O., Sborgi L., Boussofara L., Hopkins R., Robinson K., Szeverenyi I., Takeichi T., Balaji R., Lau A. (2016). Germline NLRP1 Mutations Cause Skin Inflammatory and Cancer Susceptibility Syndromes via Inflammasome Activation. Cell.

[58] Herlin T., Jorgensen S.E., Host C., Mitchell P.S., Christensen M.H., Laustsen M., Larsen D.A., Schmidt F.I., Christiansen M., Mogensen T.H. (2020). Autoinflammatory disease with corneal and mucosal dyskeratosis caused by a novel NLRP1 variant. Rheumatology.

[59] Drutman S.B., Haerynck F., Zhong F.L., Hum D., Hernandez N.J., Belkaya S., Rapaport F., de Jong S.J., Creytens D., Tavernier S.J. (2019). Homozygous NLRP1 gain-of-function mutation in siblings with a syndromic form of recurrent respiratory papillomatosis. Proc. Natl. Acad. Sci. USA.

[60] Grandemange S., Sanchez E., Louis-Plence P., Tran Mau-Them F., Bessis D., Coubes C., Frouin E., Seyger M., Girard M., Puechberty J. (2017). A new autoinflammatory and autoimmune syndrome associated with NLRP1 mutations: NAIAD (NLRP1-associated autoinflammation with arthritis and dyskeratosis). Ann. Rheum. Dis..

[61] Chui A.J., Okondo M.C., Rao S.D., Gai K., Griswold A.R., Johnson D.C., Ball D.P., Taabazuing C.Y., Orth E.L., Vittimberga B.A. (2019). N-terminal degradation activates the NLRP1B inflammasome. Science.

[62] Sandstrom A., Mitchell P.S., Goers L., Mu E.W., Lesser C.F., Vance R.E. (2019). Functional degradation: A mechanism of NLRP1 inflammasome activation by diverse pathogen enzymes. Science.

[63] Ewald S.E., Chavarria-Smith J., Boothroyd J.C. (2014). NLRP1 is an inflammasome sensor for Toxoplasma gondii. Infect. Immun..

[64] Tsu B.V., Beierschmitt C., Ryan A.P., Agarwal R., Mitchell P.S., Daugherty M.D. (2021). Diverse viral proteases activate the NLRP1 inflammasome. Elife.

[65] Robinson K.S., Teo D.E.T., Tan K.S., Toh G.A., Ong H.H., Lim C.K., Lay K., Au B.V., Lew T.S., Chu J.J.H. (2020). Enteroviral 3C protease activates the human NLRP1 inflammasome in airway epithelia. Science.

[66] Fenini G., Grossi S., Contassot E., Biedermann T., Reichmann E., French L.E., Beer H.D. (2018). Genome Editing of Human Primary Keratinocytes by CRISPR/Cas9 Reveals an Essential Role of the NLRP1 Inflammasome in UVB Sensing. J. Investig. Dermatol..

[67] Sand J., Haertel E., Biedermann T., Contassot E., Reichmann E., French L.E., Werner S., Beer H.D. (2018). Expression of inflammasome proteins and inflammasome activation occurs in human, but not in murine keratinocytes. Cell Death Dis..

[68] Robinson K.S., Toh G.A., Rozario P., Bayat S., Sun Z., Bauernfried S., Nadkarni R., Harapas C.R., Lim C.K., Chu W. (2022). Human NLRP1 is activated by ZAKɑ-driven ribotoxic stress response. bioRxiv.

[69] Jenster L.-M., Lange K.-E., Normann S., vom Hemdt A., Wuerth J.D., Schiffelers L.D.J., Tesfamariam Y.M., Gohr F.N., Klein L., Kaltheuner I.H. (2022). P38 kinases mediate NLRP1 inflammasome activation after ribotoxic stress response and virus infection. bioRxiv.

[70] Bauernfried S., Scherr M.J., Pichlmair A., Duderstadt K.E., Hornung V. (2020). Human NLRP1 is a sensor for double-stranded RNA. Science.

[71] Wang Q., Hsiao J., Yardeny N., Huang H.-C., O’Mara C.M., Orth-He E.L., Ball D.P., Bachovchin D.A. (2022). The NLRP1 and CARD8 inflammasomes detect reductive stress. bioRxiv.

[72] Orth-He E.L., Huang H.-C., Rao S.D., Wang Q., Chen Q., O’Mara C.M., Chui A.J., Saoi M., Griswold A.R., Bhattacharjee A. (2022). Cytosolic peptide accumulation activates the NLRP1 and CARD8 inflammasomes. bioRxiv.

[73] Canna S.W., de Jesus A.A., Gouni S., Brooks S.R., Marrero B., Liu Y., DiMattia M.A., Zaal K.J., Sanchez G.A., Kim H. (2014). An activating NLRC4 inflammasome mutation causes autoinflammation with recurrent macrophage activation syndrome. Nat. Genet..

[74] Kitamura A., Sasaki Y., Abe T., Kano H., Yasutomo K. (2014). An inherited mutation in NLRC4 causes autoinflammation in human and mice. J. Exp. Med..

[75] Duncan J.A., Canna S.W. (2018). The NLRC4 Inflammasome. Immunol. Rev..

[76] Andrade W.A., Zamboni D.S. (2020). NLRC4 biology in immunity and inflammation. J. Leukoc. Biol..

[77] Freeman L., Guo H., David C.N., Brickey W.J., Jha S., Ting J.P. (2017). NLR members NLRC4 and NLRP3 mediate sterile inflammasome activation in microglia and astrocytes. J. Exp. Med..

[78] Ip W.K., Medzhitov R. (2015). Macrophages monitor tissue osmolarity and induce inflammatory response through NLRP3 and NLRC4 inflammasome activation. Nat. Commun..

[79] Wang S.B., Narendran S., Hirahara S., Varshney A., Pereira F., Apicella I., Ambati M., Ambati V.L., Yerramothu P., Ambati K. (2021). DDX17 is an essential mediator of sterile NLRC4 inflammasome activation by retrotransposon RNAs. Sci. Immunol..

[80] Stetson D.B. (2012). Endogenous retroelements and autoimmune disease. Curr. Opin. Immunol..

[81] Jakobs C., Perner S., Hornung V. (2015). AIM2 Drives Joint Inflammation in a Self-DNA Triggered Model of Chronic Polyarthritis. PLoS ONE.

[82] Baum R., Sharma S., Carpenter S., Li Q.Z., Busto P., Fitzgerald K.A., Marshak-Rothstein A., Gravallese E.M. (2015). Cutting edge: AIM2 and endosomal TLRs differentially regulate arthritis and autoantibody production in DNase II-deficient mice. J. Immunol..

[83] The International FMF Consortium (1997). Ancient missense mutations in a new member of the RoRet gene family are likely to cause familial Mediterranean fever. Cell.

[84] Bernot A., Clepet C., Dasilva C., Devaud C., Petit J.-L., Caloustian C., Cruaud C., Samson D., Pulcini F., The French FMF Consortium (1997). A candidate gene for familial Mediterranean fever. Nat. Genet..

[85] Masters S.L., Lagou V., Jeru I., Baker P.J., Van Eyck L., Parry D.A., Lawless D., De Nardo D., Garcia-Perez J.E., Dagley L.F. (2016). Familial autoinflammation with neutrophilic dermatosis reveals a regulatory mechanism of pyrin activation. Sci. Transl. Med..

[86] Hagar J.A., Powell D.A., Aachoui Y., Ernst R.K., Miao E.A. (2013). Cytoplasmic LPS activates caspase-11: Implications in TLR4-independent endotoxic shock. Science.

[87] Kayagaki N., Wong M.T., Stowe I.B., Ramani S.R., Gonzalez L.C., Akashi-Takamura S., Miyake K., Zhang J., Lee W.P., Muszynski A. (2013). Noncanonical inflammasome activation by intracellular LPS independent of TLR4. Science.

[88] Zanoni I., Tan Y., Di Gioia M., Broggi A., Ruan J., Shi J., Donado C.A., Shao F., Wu H., Springstead J.R. (2016). An endogenous caspase-11 ligand elicits interleukin-1 release from living dendritic cells. Science.

[89] Kutsch M., Coers J. (2021). Human guanylate binding proteins: Nanomachines orchestrating host defense. FEBS J..

[90] Santos J.C., Boucher D., Schneider L.K., Demarco B., Dilucca M., Shkarina K., Heilig R., Chen K.W., Lim R.Y.H., Broz P. (2020). Human GBP1 binds LPS to initiate assembly of a caspase-4 activating platform on cytosolic bacteria. Nat. Commun..

[91] Wandel M.P., Kim B.H., Park E.S., Boyle K.B., Nayak K., Lagrange B., Herod A., Henry T., Zilbauer M., Rohde J. (2020). Guanylate-binding proteins convert cytosolic bacteria into caspase-4 signaling platforms. Nat. Immunol..

[92] Poelzl A., Lassnig C., Tangermann S., Hromadova D., Reichart U., Gawish R., Mueller K., Moriggl R., Linkermann A., Glosmann M. (2020). TYK2 licenses non-canonical inflammasome activation during endotoxemia. Cell Death Differ..

[93] Eren E., Planes R., Bagayoko S., Bordignon P.J., Chaoui K., Hessel A., Santoni K., Pinilla M., Lagrange B., Burlet-Schiltz O. (2020). Irgm2 and Gate-16 cooperatively dampen Gram-negative bacteria-induced caspase-11 response. EMBO Rep..

[94] Finethy R., Dockterman J., Kutsch M., Orench-Rivera N., Wallace G.D., Piro A.S., Luoma S., Haldar A.K., Hwang S., Martinez J. (2020). Dynamin-related Irgm proteins modulate LPS-induced caspase-11 activation and septic shock. EMBO Rep..

[95] Sakaguchi N., Sasai M., Bando H., Lee Y., Pradipta A., Ma J.S., Yamamoto M. (2020). Role of Gate-16 and Gabarap in Prevention of Caspase-11-Dependent Excess Inflammation and Lethal Endotoxic Shock. Front. Immunol..

[96] Rojas-Lopez M., Zajac A.S., Wood T.E., Miller K.A., Gil-Marqués M.L., Hachey A.C., Kharbanda V., Egger K.T., Goldberg M.B. (2022). Pattern Recognition Receptor for Bacterial Lipopolysaccharide in the Cytosol of Human Macrophages. bioRxiv.

[97] Monteleone M., Stow J.L., Schroder K. (2015). Mechanisms of unconventional secretion of IL-1 family cytokines. Cytokine.

[98] Tamura M., Tanaka S., Fujii T., Aoki A., Komiyama H., Ezawa K., Sumiyama K., Sagai T., Shiroishi T. (2007). Members of a novel gene family, Gsdm, are expressed exclusively in the epithelium of the skin and gastrointestinal tract in a highly tissue-specific manner. Genomics.

[99] Saeki N., Kuwahara Y., Sasaki H., Satoh H., Shiroishi T. (2000). Gasdermin (Gsdm) localizing to mouse Chromosome 11 is predominantly expressed in upper gastrointestinal tract but significantly suppressed in human gastric cancer cells. Mamm. Genome.

[100] Bourdonnay E., Henry T. (2022). Transcriptional and Epigenetic Regulation of Gasdermins. J. Mol. Biol..

[101] De Schutter E., Roelandt R., Riquet F.B., Van Camp G., Wullaert A., Vandenabeele P. (2021). Punching Holes in Cellular Membranes: Biology and Evolution of Gasdermins. Trends Cell Biol..

[102] Angosto-Bazarra D., Alarcon-Vila C., Hurtado-Navarro L., Banos M.C., Rivers-Auty J., Pelegrin P. (2022). Evolutionary analyses of the gasdermin family suggest conserved roles in infection response despite loss of pore-forming functionality. BMC Biol..

[103] Jiang S., Zhou Z., Sun Y., Zhang T., Sun L. (2020). Coral gasdermin triggers pyroptosis. Sci. Immunol..

[104] Johnson A.G., Wein T., Mayer M.L., Duncan-Lowey B., Yirmiya E., Oppenheimer-Shaanan Y., Amitai G., Sorek R., Kranzusch P.J. (2022). Bacterial gasdermins reveal an ancient mechanism of cell death. Science.

[105] Daskalov A., Gladieux P., Heller J., Glass N.L. (2019). Programmed Cell Death in Neurospora crassa Is Controlled by the Allorecognition Determinant rcd-1. Genetics.

[106] Clave C., Dyrka W., Turcotte E.A., Granger-Farbos A., Ibarlosa L., Pinson B., Vance R.E., Saupe S.J., Daskalov A. (2022). Fungal gasdermin-like proteins are controlled by proteolytic cleavage. Proc. Natl. Acad. Sci. USA.

[107] Van Rossom S., Op de Beeck K., Franssens V., Swinnen E., Schepers A., Ghillebert R., Caldara M., Van Camp G., Winderickx J. (2012). The splicing mutant of the human tumor suppressor protein DFNA5 induces programmed cell death when expressed in the yeast Saccharomyces cerevisiae. Front. Oncol..

[108] Op de Beeck K., Van Camp G., Thys S., Cools N., Callebaut I., Vrijens K., Van Nassauw L., Van Tendeloo V.F., Timmermans J.P., Van Laer L. (2011). The DFNA5 gene, responsible for hearing loss and involved in cancer, encodes a novel apoptosis-inducing protein. Eur. J. Hum. Genet..

[109] Kayagaki N., Stowe I.B., Lee B.L., O’Rourke K., Anderson K., Warming S., Cuellar T., Haley B., Roose-Girma M., Phung Q.T. (2015). Caspase-11 cleaves gasdermin D for non-canonical inflammasome signalling. Nature.

[110] Shi J., Zhao Y., Wang K., Shi X., Wang Y., Huang H., Zhuang Y., Cai T., Wang F., Shao F. (2015). Cleavage of GSDMD by inflammatory caspases determines pyroptotic cell death. Nature.

[111] He W.T., Wan H., Hu L., Chen P., Wang X., Huang Z., Yang Z.H., Zhong C.Q., Han J. (2015). Gasdermin D is an executor of pyroptosis and required for interleukin-1beta secretion. Cell Res..

[112] Wang K., Sun Q., Zhong X., Zeng M., Zeng H., Shi X., Li Z., Wang Y., Zhao Q., Shao F. (2020). Structural Mechanism for GSDMD Targeting by Autoprocessed Caspases in Py.yroptosis. Cell.

[113] Liu Z., Wang C., Yang J., Chen Y., Zhou B., Abbott D.W., Xiao T.S. (2020). Caspase-1 Engages Full-Length Gasdermin D through Two Distinct Interfaces That Mediate Caspase Recruitment and Substrate Cleavage. Immunity.

[114] Aglietti R.A., Estevez A., Gupta A., Ramirez M.G., Liu P.S., Kayagaki N., Ciferri C., Dixit V.M., Dueber E.C. (2016). GsdmD p30 elicited by caspase-11 during pyroptosis forms pores in membranes. Proc. Natl. Acad. Sci. USA.

[115] Ding J., Wang K., Liu W., She Y., Sun Q., Shi J., Sun H., Wang D.C., Shao F. (2016). Pore-forming activity and structural autoinhibition of the gasdermin family. Nature.

[116] Liu X., Zhang Z., Ruan J., Pan Y., Magupalli V.G., Wu H., Lieberman J. (2016). Inflammasome-activated gasdermin D causes pyroptosis by forming membrane pores. Nature.

[117] Sborgi L., Ruhl S., Mulvihill E., Pipercevic J., Heilig R., Stahlberg H., Farady C.J., Muller D.J., Broz P., Hiller S. (2016). GSDMD membrane pore formation constitutes the mechanism of pyroptotic cell death. EMBO J..

[118] Mulvihill E., Sborgi L., Mari S.A., Pfreundschuh M., Hiller S., Muller D.J. (2018). Mechanism of membrane pore formation by human gasdermin-D. EMBO J..

[119] Santa Cruz Garcia A.B., Schnur K.P., Malik A.B., Mo G.C.H. (2022). Gasdermin D pores are dynamically regulated by local phosphoinositide circuitry. Nat. Commun..

[120] Heilig R., Dick M.S., Sborgi L., Meunier E., Hiller S., Broz P. (2018). The Gasdermin-D pore acts as a conduit for IL-1beta secretion in mice. Eur. J. Immunol..

[121] Evavold C.L., Ruan J., Tan Y., Xia S., Wu H., Kagan J.C. (2018). The Pore-Forming Protein Gasdermin D Regulates Interleukin-1 Secretion from Living Macrophages. Immunity.

[122] Xia S., Zhang Z., Magupalli V.G., Pablo J.L., Dong Y., Vora S.M., Wang L., Fu T.M., Jacobson M.P., Greka A. (2021). Gasdermin D pore structure reveals preferential release of mature interleukin-1. Nature.

[123] Xie W.J., Xia S., Warshel A., Wu H. (2022). Electrostatic influence on IL-1 transport through the GSDMD pore. Proc. Natl. Acad. Sci. USA.

[124] Monteleone M., Stanley A.C., Chen K.W., Brown D.L., Bezbradica J.S., von Pein J.B., Holley C.L., Boucher D., Shakespear M.R., Kapetanovic R. (2018). Interleukin-1beta Maturation Triggers Its Relocation to the Plasma Membrane for Gasdermin-D-Dependent and -Independent Secretion. Cell Rep..

[125] Ruan J., Xia S., Liu X., Lieberman J., Wu H. (2018). Cryo-EM structure of the gasdermin A3 membrane pore. Nature.

[126] Kayagaki N., Kornfeld O.S., Lee B.L., Stowe I.B., O’Rourke K., Li Q., Sandoval W., Yan D., Kang J., Xu M. (2021). NINJ1 mediates plasma membrane rupture during lytic cell death. Nature.

[127] Borges J.P., Sætra R.S.R., Volchuk A., Bugge M., Kilburn B., Goldenberg N.M., Flo T.H., Steinberg B.E. (2022). Glycine targets NINJ1-mediated plasma membrane rupture to provide cytoprotection. BioRxiv.

[128] Ahn B.J., Le H., Shin M.W., Bae S.J., Lee E.J., Wee H.J., Cha J.H., Lee H.J., Lee H.S., Kim J.H. (2014). Ninjurin1 deficiency attenuates susceptibility of experimental autoimmune encephalomyelitis in mice. J. Biol. Chem..

[129] Choi S., Woo J.K., Jang Y.S., Kang J.H., Hwang J.I., Seong J.K., Yoon Y.S., Oh S.H. (2018). Ninjurin1 Plays a Crucial Role in Pulmonary Fibrosis by Promoting Interaction between Macrophages and Alveolar Epithelial Cells. Sci. Rep..

[130] Galluzzi L., Vitale I., Aaronson S.A., Abrams J.M., Adam D., Agostinis P., Alnemri E.S., Altucci L., Amelio I., Andrews D.W. (2018). Molecular mechanisms of cell death: Recommendations of the Nomenclature Committee on Cell Death 2018. Cell Death Differ..

[131] Shi J., Gao W., Shao F. (2017). Pyroptosis: Gasdermin-Mediated Programmed Necrotic Cell Death. Trends Biochem. Sci..

[132] Chen K.W., Monteleone M., Boucher D., Sollberger G., Ramnath D., Condon N.D., von Pein J.B., Broz P., Sweet M.J., Schroder K. (2018). Noncanonical inflammasome signaling elicits gasdermin D-dependent neutrophil extracellular traps. Sci. Immunol..

[133] Kambara H., Liu F., Zhang X., Liu P., Bajrami B., Teng Y., Zhao L., Zhou S., Yu H., Zhou W. (2018). Gasdermin D Exerts Anti-inflammatory Effects by Promoting Neutrophil Death. Cell Rep..

[134] Sollberger G., Choidas A., Burn G.L., Habenberger P., Di Lucrezia R., Kordes S., Menninger S., Eickhoff J., Nussbaumer P., Klebl B. (2018). Gasdermin D plays a vital role in the generation of neutrophil extracellular traps. Sci. Immunol..

[135] Kayagaki N., Lee B.L., Stowe I.B., Kornfeld O.S., O’Rourke K., Mirrashidi K.M., Haley B., Watanabe C., Roose-Girma M., Modrusan Z. (2019). IRF2 transcriptionally induces GSDMD expression for pyroptosis. Sci. Signal..

[136] Benaoudia S., Martin A., Puig Gamez M., Gay G., Lagrange B., Cornut M., Krasnykov K., Claude J.B., Bourgeois C.F., Hughes S. (2019). A genome-wide screen identifies IRF2 as a key regulator of caspase-4 in human cells. EMBO Rep..

[137] Li Y., Guo X., Hu C., Du Y., Guo C., Di W., Zhao W., Huang G., Li C., Lu Q. (2018). Type I IFN operates pyroptosis and necroptosis during multidrug-resistant A. baumannii infection. Cell Death Differ..

[138] Shi Y., Yang Y., Xu W., Shi D., Xu W., Fu X., Lv Q., Xia J., Shi F. (2022). E3 ubiquitin ligase SYVN1 is a key positive regulator for GSDMD-mediated pyroptosis. Cell Death Dis..

[139] Rogers C., Erkes D.A., Nardone A., Aplin A.E., Fernandes-Alnemri T., Alnemri E.S. (2019). Gasdermin pores permeabilize mitochondria to augment caspase-3 activation during apoptosis and inflammasome activation. Nat. Commun..

[140] Humphries F., Shmuel-Galia L., Ketelut-Carneiro N., Li S., Wang B., Nemmara V.V., Wilson R., Jiang Z., Khalighinejad F., Muneeruddin K. (2020). Succination inactivates gasdermin D and blocks pyroptosis. Science.

[141] Bambouskova M., Potuckova L., Paulenda T., Kerndl M., Mogilenko D.A., Lizotte K., Swain A., Hayes S., Sheldon R.D., Kim H. (2021). Itaconate confers tolerance to late NLRP3 inflammasome activation. Cell Rep..

[142] Wang Y., Shi P., Chen Q., Huang Z., Zou D., Zhang J., Gao X., Lin Z. (2019). Mitochondrial ROS promote macrophage pyroptosis by inducing GSDMD oxidation. J. Mol. Cell Biol..

[143] Hu L., Chen M., Chen X., Zhao C., Fang Z., Wang H., Dai H. (2020). Chemotherapy-induced pyroptosis is mediated by BAK/BAX-caspase-3-GSDME pathway and inhibited by 2-bromopalmitate. Cell Death Dis..

[144] Devant P., Boršić E., Ngwa E.M., Thiagarajah J.R., Hafner-Bratkovič I., Evavold C.L., Kagan J.C. (2022). The pore-forming protein gasdermin D is a cellular redox sensor. bioRxiv.

[145] Gao W., Li Y., Liu X., Wang S., Mei P., Chen Z., Liu K., Li S., Xu X.W., Gan J. (2022). TRIM21 regulates pyroptotic cell death by promoting Gasdermin D oligomerization. Cell Death Differ..

[146] Liu G.Y., Sabatini D.M. (2020). mTOR at the nexus of nutrition, growth, ageing and disease. Nat. Rev. Mol. Cell Biol..

[147] Evavold C.L., Hafner-Bratkovic I., Devant P., D’Andrea J.M., Ngwa E.M., Borsic E., Doench J.G., LaFleur M.W., Sharpe A.H., Thiagarajah J.R. (2021). Control of gasdermin D oligomerization and pyroptosis by the Ragulator-Rag-mTORC1 pathway. Cell.

[148] Taabazuing C.Y., Okondo M.C., Bachovchin D.A. (2017). Pyroptosis and Apoptosis Pathways Engage in Bidirectional Crosstalk in Monocytes and Macrophages. Cell Chem. Biol..

[149] Chen K.W., Demarco B., Heilig R., Shkarina K., Boettcher A., Farady C.J., Pelczar P., Broz P. (2019). Extrinsic and intrinsic apoptosis activate pannexin-1 to drive NLRP3 inflammasome assembly. EMBO J..

[150] Orning P., Weng D., Starheim K., Ratner D., Best Z., Lee B., Brooks A., Xia S., Wu H., Kelliher M.A. (2018). Pathogen blockade of TAK1 triggers caspase-8-dependent cleavage of gasdermin D and cell death. Science.

[151] Sanjo H., Nakayama J., Yoshizawa T., Fehling H.J., Akira S., Taki S. (2019). Cutting Edge: TAK1 Safeguards Macrophages against Proinflammatory Cell Death. J. Immunol..

[152] Sarhan J., Liu B.C., Muendlein H.I., Li P., Nilson R., Tang A.Y., Rongvaux A., Bunnell S.C., Shao F., Green D.R. (2018). Caspase-8 induces cleavage of gasdermin D to elicit pyroptosis during Yersinia infection. Proc. Natl. Acad. Sci. USA.

[153] Jimenez A.J., Maiuri P., Lafaurie-Janvore J., Divoux S., Piel M., Perez F. (2014). ESCRT machinery is required for plasma membrane repair. Science.

[154] Skowyra M.L., Schlesinger P.H., Naismith T.V., Hanson P.I. (2018). Triggered recruitment of ESCRT machinery promotes endolysosomal repair. Science.

[155] Gong Y.N., Guy C., Olauson H., Becker J.U., Yang M., Fitzgerald P., Linkermann A., Green D.R. (2017). ESCRT-III Acts Downstream of MLKL to Regulate Necroptotic Cell Death and Its Consequences. Cell.

[156] Ruhl S., Shkarina K., Demarco B., Heilig R., Santos J.C., Broz P. (2018). ESCRT-dependent membrane repair negatively regulates pyroptosis downstream of GSDMD activation. Science.

[157] Burgener S.S., Leborgne N.G.F., Snipas S.J., Salvesen G.S., Bird P.I., Benarafa C. (2019). Cathepsin G Inhibition by Serpinb1 and Serpinb6 Prevents Programmed Necrosis in Neutrophils and Monocytes and Reduces GSDMD-Driven Inflammation. Cell Rep..

[158] Yamaoka Y., Matsunaga S., Jeremiah S.S., Nishi M., Miyakawa K., Morita T., Khatun H., Shimizu H., Okabe N., Kimura H. (2021). Zika virus protease induces caspase-independent pyroptotic cell death by directly cleaving gasdermin D. Biochem. Biophys. Res. Commun..

[159] Lei X., Zhang Z., Xiao X., Qi J., He B., Wang J. (2017). Enterovirus 71 Inhibits Pyroptosis through Cleavage of Gasdermin D. J. Virol..

[160] Ma J., Zhu F., Zhao M., Shao F., Yu D., Ma J., Zhang X., Li W., Qian Y., Zhang Y. (2021). SARS-CoV-2 nucleocapsid suppresses host pyroptosis by blocking Gasdermin D cleavage. EMBO J..

[161] Broz P., Pelegrin P., Shao F. (2020). The gasdermins, a protein family executing cell death and inflammation. Nat. Rev. Immunol..

[162] Deng W., Bai Y., Deng F., Pan Y., Mei S., Zheng Z., Min R., Wu Z., Li W., Miao R. (2022). Streptococcal pyrogenic exotoxin B cleaves GSDMA and triggers pyroptosis. Nature.

[163] Zhou Z., He H., Wang K., Shi X., Wang Y., Su Y., Wang Y., Li D., Liu W., Zhang Y. (2020). Granzyme A from cytotoxic lymphocytes cleaves GSDMB to trigger pyroptosis in target cells. Science.

[164] Chao K.L., Kulakova L., Herzberg O. (2017). Gene polymorphism linked to increased asthma and IBD risk alters gasdermin-B structure, a sulfatide and phosphoinositide binding protein. Proc. Natl. Acad. Sci. USA.

[165] Chen Q., Shi P., Wang Y., Zou D., Wu X., Wang D., Hu Q., Zou Y., Huang Z., Ren J. (2019). GSDMB promotes non-canonical pyroptosis by enhancing caspase-4 activity. J. Mol. Cell Biol..

[166] Hansen J.M., de Jong M.F., Wu Q., Zhang L.S., Heisler D.B., Alto L.T., Alto N.M. (2021). Pathogenic ubiquitination of GSDMB inhibits NK cell bactericidal functions. Cell.

[167] Luchetti G., Roncaioli J.L., Chavez R.A., Schubert A.F., Kofoed E.M., Reja R., Cheung T.K., Liang Y., Webster J.D., Lehoux I. (2021). Shigella ubiquitin ligase IpaH7.8 targets gasdermin D for degradation to prevent pyroptosis and enable infection. Cell Host Microbe.

[168] Hou J., Zhao R., Xia W., Chang C.W., You Y., Hsu J.M., Nie L., Chen Y., Wang Y.C., Liu C. (2020). PD-L1-mediated gasdermin C expression switches apoptosis to pyroptosis in cancer cells and facilitates tumour necrosis. Nat. Cell Biol..

[169] Zhang J.Y., Zhou B., Sun R.Y., Ai Y.L., Cheng K., Li F.N., Wang B.R., Liu F.J., Jiang Z.H., Wang W.J. (2021). The metabolite alpha-KG induces GSDMC-dependent pyroptosis through death receptor 6-activated caspase-8. Cell Res..

[170] Xi R., Montague J., Lin X., Lu C., Lei W., Tanaka K., Zhang Y.V., Xu X., Zheng X., Zhou X. (2021). Up-regulation of gasdermin C in mouse small intestine is associated with lytic cell death in enterocytes in worm-induced type 2 immunity. Proc. Natl. Acad. Sci. USA.

[171] Sposito B., Mambu J., Gwilt K.B., Spinelli L., Andreeva N., Galland F., Naquet P., Mitsialis V., Thiagarajah J.R., Snapper S.B. (2022). Type III interferons induce pyroptosis in gut epithelial cells and delay tissue restitution upon acute intestinal injury. bioRxiv.

[172] Zhao M., Ren K., Xiong X., Xin Y., Zou Y., Maynard J.C., Kim A., Battist A.P., Koneripalli N., Wang Y. (2022). Epithelial STAT6 O-GlcNAcylation drives a concerted anti-helminth alarmin response dependent on tuft cell hyperplasia and Gasdermin C. Immunity.

[173] Wang Y., Gao W., Shi X., Ding J., Liu W., He H., Wang K., Shao F. (2017). Chemotherapy drugs induce pyroptosis through caspase-3 cleavage of a gasdermin. Nature.

[174] Rogers C., Fernandes-Alnemri T., Mayes L., Alnemri D., Cingolani G., Alnemri E.S. (2017). Cleavage of DFNA5 by caspase-3 during apoptosis mediates progression to secondary necrotic/pyroptotic cell death. Nat. Commun..

[175] Zhang Z., Zhang Y., Xia S., Kong Q., Li S., Liu X., Junqueira C., Meza-Sosa K.F., Mok T.M.Y., Ansara J. (2020). Gasdermin E suppresses tumour growth by activating anti-tumour immunity. Nature.

[176] Liu Y., Fang Y., Chen X., Wang Z., Liang X., Zhang T., Liu M., Zhou N., Lv J., Tang K. (2020). Gasdermin E-mediated target cell pyroptosis by CAR T cells triggers cytokine release syndrome. Sci. Immunol..

[177] Tan G., Huang C., Chen J., Chen B., Zhi F. (2021). Gasdermin-E-mediated pyroptosis participates in the pathogenesis of Crohn’s disease by promoting intestinal inflammation. Cell Rep..

[178] Chen K.W., Demarco B., Ramos S., Heilig R., Goris M., Grayczyk J.P., Assenmacher C.A., Radaelli E., Joannas L.D., Henao-Mejia J. (2021). RIPK1 activates distinct gasdermins in macrophages and neutrophils upon pathogen blockade of innate immune signaling. Proc. Natl. Acad. Sci. USA.

[179] Orzalli M.H., Prochera A., Payne L., Smith A., Garlick J.A., Kagan J.C. (2021). Virus-mediated inactivation of anti-apoptotic Bcl-2 family members promotes Gasdermin-E-dependent pyroptosis in barrier epithelial cells. Immunity.

[180] De Schutter E., Ramon J., Pfeuty B., De Tender C., Stremersch S., Raemdonck K., de Beeck K.O., Declercq W., Riquet F.B., Braeckmans K. (2021). Plasma membrane perforation by GSDME during apoptosis-driven secondary necrosis. Cell Mol. Life Sci..

[181] Rana N., Privitera G., Kondolf H.C., Bulek K., Lechuga S., De Salvo C., Corridoni D., Antanaviciute A., Maywald R.L., Hurtado A.M. (2022). GSDMB is increased in IBD and regulates epithelial restitution/repair independent of pyroptosis. Cell.

[182] Bulek K., Zhao J., Liao Y., Rana N., Corridoni D., Antanaviciute A., Chen X., Wang H., Qian W., Miller-Little W.A. (2020). Epithelial-derived gasdermin D mediates nonlytic IL-1beta release during experimental colitis. J. Clin. Investig..

[183] Hoffman H.M., Throne M.L., Amar N.J., Sebai M., Kivitz A.J., Kavanaugh A., Weinstein S.P., Belomestnov P., Yancopoulos G.D., Stahl N. (2008). Efficacy and safety of rilonacept (interleukin-1 Trap) in patients with cryopyrin-associated periodic syndromes: Results from two sequential placebo-controlled studies. Arthritis Rheum..

[184] Lachmann H.J., Lowe P., Felix S.D., Rordorf C., Leslie K., Madhoo S., Wittkowski H., Bek S., Hartmann N., Bosset S. (2009). In vivo regulation of interleukin 1beta in patients with cryopyrin-associated periodic syndromes. J. Exp. Med..

[185] Xiao J., Wang C., Yao J.C., Alippe Y., Xu C., Kress D., Civitelli R., Abu-Amer Y., Kanneganti T.D., Link D.C. (2018). Gasdermin D mediates the pathogenesis of neonatal-onset multisystem inflammatory disease in mice. PLoS Biol..

[186] Kanneganti A., Malireddi R.K.S., Saavedra P.H.V., Vande Walle L., Van Gorp H., Kambara H., Tillman H., Vogel P., Luo H.R., Xavier R.J. (2018). GSDMD is critical for autoinflammatory pathology in a mouse model of Familial Mediterranean Fever. J. Exp. Med..

[187] Li S., Wu Y., Yang D., Wu C., Ma C., Liu X., Moynagh P.N., Wang B., Hu G., Yang S. (2019). Gasdermin D in peripheral myeloid cells drives neuroinflammation in experimental autoimmune encephalomyelitis. J. Exp. Med..

[188] McKenzie B.A., Mamik M.K., Saito L.B., Boghozian R., Monaco M.C., Major E.O., Lu J.Q., Branton W.G., Power C. (2018). Caspase-1 inhibition prevents glial inflammasome activation and pyroptosis in models of multiple sclerosis. Proc. Natl. Acad. Sci. USA.

[189] Jiang K., Tu Z., Chen K., Xu Y., Chen F., Xu S., Shi T., Qian J., Shen L., Hwa J. (2022). Gasdermin D inhibition confers antineutrophil-mediated cardioprotection in acute myocardial infarction. J. Clin. Investig..

[190] Kang R., Zeng L., Zhu S., Xie Y., Liu J., Wen Q., Cao L., Xie M., Ran Q., Kroemer G. (2018). Lipid Peroxidation Drives Gasdermin D-Mediated Pyroptosis in Lethal Polymicrobial Sepsis. Cell Host Microbe.

[191] Chen H., Li Y., Wu J., Li G., Tao X., Lai K., Yuan Y., Zhang X., Zou Z., Xu Y. (2020). RIPK3 collaborates with GSDMD to drive tissue injury in lethal polymicrobial sepsis. Cell Death Differ..

[192] Wang X., Blanco L.P., Carmona-Rivera C., Nakabo S., Pedersen H.L., Yu Z.X., Kaplan M.J. (2020). Effects of Gasdermin D in Modulating Murine Lupus and its Associated Organ Damage. Arthritis Rheumatol..

[193] Ma C., Yang D., Wang B., Wu C., Wu Y., Li S., Liu X., Lassen K., Dai L., Yang S. (2020). Gasdermin D in macrophages restrains colitis by controlling cGAS-mediated inflammation. Sci. Adv..

[194] Gao H., Cao M., Yao Y., Hu W., Sun H., Zhang Y., Zeng C., Tang J., Luan S., Chen P. (2021). Dysregulated Microbiota-Driven Gasdermin D Activation Promotes Colitis Development by Mediating IL-18 Release. Front. Immunol..

[195] Wu C., Lu W., Zhang Y., Zhang G., Shi X., Hisada Y., Grover S.P., Zhang X., Li L., Xiang B. (2019). Inflammasome Activation Triggers Blood Clotting and Host Death through Pyroptosis. Immunity.

[196] Yang X., Cheng X., Tang Y., Qiu X., Wang Y., Kang H., Wu J., Wang Z., Liu Y., Chen F. (2019). Bacterial Endotoxin Activates the Coagulation Cascade through Gasdermin D-Dependent Phosphatidylserine Exposure. Immunity.

[197] Zhang H., Zeng L., Xie M., Liu J., Zhou B., Wu R., Cao L., Kroemer G., Wang H., Billiar T.R. (2020). TMEM173 Drives Lethal Coagulation in Sepsis. Cell Host Microbe.

[198] Zhang J., Yu Q., Jiang D., Yu K., Yu W., Chi Z., Chen S., Li M., Yang D., Wang Z. (2022). Epithelial Gasdermin D shapes the host-microbial interface by driving mucus layer formation. Sci. Immunol..

[199] Cerqueira D.M., Gomes M.T.R., Silva A.L.N., Rungue M., Assis N.R.G., Guimaraes E.S., Morais S.B., Broz P., Zamboni D.S., Oliveira S.C. (2018). Guanylate-binding protein 5 licenses caspase-11 for Gasdermin-D mediated host resistance to Brucella abortus infection. PLoS Pathog..

[200] Schneider K.S., Gross C.J., Dreier R.F., Saller B.S., Mishra R., Gorka O., Heilig R., Meunier E., Dick M.S., Cikovic T. (2017). The Inflammasome Drives GSDMD-Independent Secondary Pyroptosis and IL-1 Release in the Absence of Caspase-1 Protease Activity. Cell Rep..

[201] Zhu Q., Zheng M., Balakrishnan A., Karki R., Kanneganti T.D. (2018). Gasdermin D Promotes AIM2 Inflammasome Activation and Is Required for Host Protection against Francisella novicida. J. Immunol..

[202] Estfanous S., Krause K., Anne M.N.K., Eltobgy M., Caution K., Abu Khweek A., Hamilton K., Badr A., Daily K., Carafice C. (2021). Gasdermin D restricts Burkholderia cenocepacia infection in vitro and in vivo. Sci. Rep..

[203] Goncalves A.V., Margolis S.R., Quirino G.F.S., Mascarenhas D.P.A., Rauch I., Nichols R.D., Ansaldo E., Fontana M.F., Vance R.E., Zamboni D.S. (2019). Gasdermin-D and Caspase-7 are the key Caspase-1/8 substrates downstream of the NAIP5/NLRC4 inflammasome required for restriction of Legionella pneumophila. PLoS Pathog..

[204] Zhu S., Ding S., Wang P., Wei Z., Pan W., Palm N.W., Yang Y., Yu H., Li H.B., Wang G. (2017). Nlrp9b inflammasome restricts rotavirus infection in intestinal epithelial cells. Nature.

[205] Dubois H., Sorgeloos F., Sarvestani S.T., Martens L., Saeys Y., Mackenzie J.M., Lamkanfi M., van Loo G., Goodfellow I., Wullaert A. (2019). Nlrp3 inflammasome activation and Gasdermin D-driven pyroptosis are immunopathogenic upon gastrointestinal norovirus infection. PLoS Pathog..

[206] Batista S.J., Still K.M., Johanson D., Thompson J.A., O’Brien C.A., Lukens J.R., Harris T.H. (2020). Gasdermin-D-dependent IL-1alpha release from microglia promotes protective immunity during chronic Toxoplasma gondii infection. Nat. Commun..

[207] Liu Z.Z., Yang Y.J., Zhou F.H., Ma K., Lin X.Q., Yan S.Q., Gao Y., Chen W. (2021). GSDMD contributes to host defence against Staphylococcus aureus skin infection by suppressing the Cxcl1-Cxcr2 axis. Vet. Res..

[208] Banerjee I., Behl B., Mendonca M., Shrivastava G., Russo A.J., Menoret A., Ghosh A., Vella A.T., Vanaja S.K., Sarkar S.N. (2018). Gasdermin D Restrains Type I Interferon Response to Cytosolic DNA by Disrupting Ionic Homeostasis. Immunity.

[209] Kaufmann T., Jost P.J., Pellegrini M., Puthalakath H., Gugasyan R., Gerondakis S., Cretney E., Smyth M.J., Silke J., Hakem R. (2009). Fatal hepatitis mediated by tumor necrosis factor TNFalpha requires caspase-8 and involves the BH3-only proteins Bid and Bim. Immunity.

[210] Duprez L., Takahashi N., Van Hauwermeiren F., Vandendriessche B., Goossens V., Vanden Berghe T., Declercq W., Libert C., Cauwels A., Vandenabeele P. (2011). RIP kinase-dependent necrosis drives lethal systemic inflammatory response syndrome. Immunity.

[211] Demarco B., Grayczyk J.P., Bjanes E., Le Roy D., Tonnus W., Assenmacher C.A., Radaelli E., Fettrelet T., Mack V., Linkermann A. (2020). Caspase-8-dependent gasdermin D cleavage promotes antimicrobial defense but confers susceptibility to TNF-induced lethality. Sci. Adv..

[212] Smolen J.S., Aletaha D., McInnes I.B. (2016). Rheumatoid arthritis. Lancet.

[213] Weyand C.M., Goronzy J.J. (2021). The immunology of rheumatoid arthritis. Nat. Immunol..

[214] Kemble S., Croft A.P. (2021). Critical Role of Synovial Tissue-Resident Macrophage and Fibroblast Subsets in the Persistence of Joint Inflammation. Front. Immunol..

[215] Culemann S., Gruneboom A., Nicolas-Avila J.A., Weidner D., Lammle K.F., Rothe T., Quintana J.A., Kirchner P., Krljanac B., Eberhardt M. (2019). Locally renewing resident synovial macrophages provide a protective barrier for the joint. Nature.

[216] Croft A.P., Campos J., Jansen K., Turner J.D., Marshall J., Attar M., Savary L., Wehmeyer C., Naylor A.J., Kemble S. (2019). Distinct fibroblast subsets drive inflammation and damage in arthritis. Nature.

[217] Matsuo Y., Mizoguchi F., Saito T., Kawahata K., Ueha S., Matsushima K., Inagaki Y., Miyasaka N., Kohsaka H. (2016). Local fibroblast proliferation but not influx is responsible for synovial hyperplasia in a murine model of rheumatoid arthritis. Biochem. Biophys. Res. Commun..

[218] Mizoguchi F., Slowikowski K., Wei K., Marshall J.L., Rao D.A., Chang S.K., Nguyen H.N., Noss E.H., Turner J.D., Earp B.E. (2018). Functionally distinct disease-associated fibroblast subsets in rheumatoid arthritis. Nat. Commun..

[219] Misharin A.V., Cuda C.M., Saber R., Turner J.D., Gierut A.K., Haines G.K., Berdnikovs S., Filer A., Clark A.R., Buckley C.D. (2014). Nonclassical Ly6C(-) monocytes drive the development of inflammatory arthritis in mice. Cell Rep..

[220] Mangan M.S.J., Olhava E.J., Roush W.R., Seidel H.M., Glick G.D., Latz E. (2018). Targeting the NLRP3 inflammasome in inflammatory diseases. Nat. Rev. Drug Discov..

[221] Fusco R., Siracusa R., Genovese T., Cuzzocrea S., Di Paola R. (2020). Focus on the Role of NLRP3 Inflammasome in Diseases. Int. J. Mol. Sci..

[222] Zhang Y., Zheng Y., Li H. (2016). NLRP3 Inflammasome Plays an Important Role in the Pathogenesis of Collagen-Induced Arthritis. Mediat. Inflamm..

[223] Jenko B., Praprotnik S., Tomsic M., Dolzan V. (2016). NLRP3 and CARD8 Polymorphisms Influence Higher Disease Activity in Rheumatoid Arthritis. J. Med. Biochem..

[224] Burska A., Boissinot M., Ponchel F. (2014). Cytokines as biomarkers in rheumatoid arthritis. Mediat. Inflamm..

[225] Kolly L., Busso N., Palmer G., Talabot-Ayer D., Chobaz V., So A. (2010). Expression and function of the NALP3 inflammasome in rheumatoid synovium. Immunology.

[226] Rosengren S., Hoffman H.M., Bugbee W., Boyle D.L. (2005). Expression and regulation of cryopyrin and related proteins in rheumatoid arthritis synovium. Ann. Rheum. Dis..

[227] Martinon F., Petrilli V., Mayor A., Tardivel A., Tschopp J. (2006). Gout-associated uric acid crystals activate the NALP3 inflammasome. Nature.

[228] So A., De Smedt T., Revaz S., Tschopp J. (2007). A pilot study of IL-1 inhibition by anakinra in acute gout. Arthritis Res. Ther..

[229] So A., De Meulemeester M., Pikhlak A., Yucel A.E., Richard D., Murphy V., Arulmani U., Sallstig P., Schlesinger N. (2010). Canakinumab for the treatment of acute flares in difficult-to-treat gouty arthritis: Results of a multicenter, phase II, dose-ranging study. Arthritis Rheum..

[230] Schlesinger N., De Meulemeester M., Pikhlak A., Yucel A.E., Richard D., Murphy V., Arulmani U., Sallstig P., So A. (2011). Canakinumab relieves symptoms of acute flares and improves health-related quality of life in patients with difficult-to-treat Gouty Arthritis by suppressing inflammation: Results of a randomized, dose-ranging study. Arthritis Res. Ther..

[231] Terkeltaub R., Sundy J.S., Schumacher H.R., Murphy F., Bookbinder S., Biedermann S., Wu R., Mellis S., Radin A. (2009). The interleukin 1 inhibitor rilonacept in treatment of chronic gouty arthritis: Results of a placebo-controlled, monosequence crossover, non-randomised, single-blind pilot study. Ann. Rheum. Dis..

[232] Ippagunta S.K., Brand D.D., Luo J., Boyd K.L., Calabrese C., Stienstra R., Van de Veerdonk F.L., Netea M.G., Joosten L.A., Lamkanfi M. (2010). Inflammasome-independent role of apoptosis-associated speck-like protein containing a CARD (ASC) in T cell priming is critical for collagen-induced arthritis. J. Biol. Chem..

[233] Kolly L., Karababa M., Joosten L.A., Narayan S., Salvi R., Petrilli V., Tschopp J., van den Berg W.B., So A.K., Busso N. (2009). Inflammatory role of ASC in antigen-induced arthritis is independent of caspase-1, NALP-3, and IPAF. J. Immunol..

[234] Yamazaki H., Takeoka M., Kitazawa M., Ehara T., Itano N., Kato H., Taniguchi S. (2012). ASC plays a role in the priming phase of the immune response to type II collagen in collagen-induced arthritis. Rheumatol. Int..

[235] Guo C., Fu R., Wang S., Huang Y., Li X., Zhou M., Zhao J., Yang N. (2018). NLRP3 inflammasome activation contributes to the pathogenesis of rheumatoid arthritis. Clin. Exp. Immunol..

[236] Matmati M., Jacques P., Maelfait J., Verheugen E., Kool M., Sze M., Geboes L., Louagie E., Mc Guire C., Vereecke L. (2011). A20 (TNFAIP3) deficiency in myeloid cells triggers erosive polyarthritis resembling rheumatoid arthritis. Nat. Genet..

[237] Vande Walle L., Van Opdenbosch N., Jacques P., Fossoul A., Verheugen E., Vogel P., Beyaert R., Elewaut D., Kanneganti T.D., van Loo G. (2014). Negative regulation of the NLRP3 inflammasome by A20 protects against arthritis. Nature.

[238] Monach P., Hattori K., Huang H., Hyatt E., Morse J., Nguyen L., Ortiz-Lopez A., Wu H.J., Mathis D., Benoist C. (2007). The K/BxN mouse model of inflammatory arthritis: Theory and practice. Methods Mol. Med..

[239] Monach P.A., Mathis D., Benoist C. (2008). The K/BxN arthritis model. Curr. Protoc. Immunol..

[240] Yang T., Sun K., Wang C., Swarnkar G., Quan S., Kress D., Xiao J., Alippe Y., Zheng H., Brophy R.H. (2021). Gasdermin D deficiency attenuates arthritis induced by traumatic injury but not autoantibody-assembled immune complexes. Arthritis Res. Ther..

[241] Zhou K., Bian C., Gu H., Cheng X., Huang Z., Chen G., Xu J., Yin X. (2021). GSDMD-dependent neutrophil extracellular traps formation contributes to fibroblast-like synoviocyte activation in rheumatoid arthritis. Res. Sq..

[242] Hu J.J., Liu X., Xia S., Zhang Z., Zhang Y., Zhao J., Ruan J., Luo X., Lou X., Bai Y. (2020). FDA-approved disulfiram inhibits pyroptosis by blocking gasdermin D pore formation. Nat. Immunol..

[243] Wang C., Yang T., Xiao J., Xu C., Alippe Y., Sun K., Kanneganti T.D., Monahan J.B., Abu-Amer Y., Lieberman J. (2021). NLRP3 inflammasome activation triggers gasdermin D-independent inflammation. Sci. Immunol..

[244] Nobel C.S., Kimland M., Nicholson D.W., Orrenius S., Slater A.F. (1997). Disulfiram is a potent inhibitor of proteases of the caspase family. Chem. Res. Toxicol..

[245] Yip N.C., Fombon I.S., Liu P., Brown S., Kannappan V., Armesilla A.L., Xu B., Cassidy J., Darling J.L., Wang W. (2011). Disulfiram modulated ROS-MAPK and NFkappaB pathways and targeted breast cancer cells with cancer stem cell-like properties. Br. J. Cancer.

[246] Wang W., McLeod H.L., Cassidy J. (2003). Disulfiram-mediated inhibition of NF-kappaB activity enhances cytotoxicity of 5-fluorouracil in human colorectal cancer cell lines. Int. J. Cancer.

[247] Celik O., Ersahin A., Acet M., Celik N., Baykus Y., Deniz R., Ozerol E., Ozerol I. (2016). Disulfiram, as a candidate NF-kappaB and proteasome inhibitor, prevents endometriotic implant growing in a rat model of endometriosis. Eur. Rev. Med. Pharmacol. Sci..

[248] Liu P., Wang Z., Brown S., Kannappan V., Tawari P.E., Jiang W., Irache J.M., Tang J.Z., Armesilla A.L., Darling J.L. (2014). Liposome encapsulated Disulfiram inhibits NFkappaB pathway and targets breast cancer stem cells in vitro and in vivo. Oncotarget.

[249] Guo X., Xu B., Pandey S., Goessl E., Brown J., Armesilla A.L., Darling J.L., Wang W. (2010). Disulfiram/copper complex inhibiting NFkappaB activity and potentiating cytotoxic effect of gemcitabine on colon and breast cancer cell lines. Cancer Lett..

[250] Adrover J.M., Carrau L., Dassler-Plenker J., Bram Y., Chandar V., Houghton S., Redmond D., Merrill J.R., Shevik M., tenOever B.R. (2022). Disulfiram inhibits neutrophil extracellular trap formation protecting rodents from acute lung injury and SARS-CoV-2 infection. JCI Insight.

[251] Zhai Z., Yang F., Xu W., Han J., Luo G., Li Y., Zhuang J., Jie H., Li X., Shi X. (2021). Attenuation of Rheumatoid Arthritis Through the Inhibition of Tumor Necrosis Factor-Induced Caspase 3/Gasdermin E-Mediated Pyroptosis. Arthritis Rheumatol..

[252] Jiang Q., Wang X., Huang E., Wang Q., Wen C., Yang G., Lu L., Cui D. (2021). Inflammasome and Its Therapeutic Targeting in Rheumatoid Arthritis. Front. Immunol..

[253] Ryder C.B., Kondolf H.C., O’Keefe M.E., Zhou B., Abbott D.W. (2022). Chemical Modulation of Gasdermin-Mediated Pyroptosis and Therapeutic Potential. J. Mol. Biol..

[254] McCoy S.S., Stannard J., Kahlenberg J.M. (2016). Targeting the inflammasome in rheumatic diseases. Transl. Res..

[255] Sun L., Wang H., Wang Z., He S., Chen S., Liao D., Wang L., Yan J., Liu W., Lei X. (2012). Mixed lineage kinase domain-like protein mediates necrosis signaling downstream of RIP3 kinase. Cell.

[256] Rathkey J.K., Zhao J., Liu Z., Chen Y., Yang J., Kondolf H.C., Benson B.L., Chirieleison S.M., Huang A.Y., Dubyak G.R. (2018). Chemical disruption of the pyroptotic pore-forming protein gasdermin D inhibits inflammatory cell death and sepsis. Sci. Immunol..

[257] Martin-Sanchez F., Diamond C., Zeitler M., Gomez A.I., Baroja-Mazo A., Bagnall J., Spiller D., White M., Daniels M.J., Mortellaro A. (2016). Inflammasome-dependent IL-1beta release depends upon membrane permeabilisation. Cell Death Differ..

[258] De Torre-Minguela C., Gomez A.I., Couillin I., Pelegrin P. (2021). Gasdermins mediate cellular release of mitochondrial DNA during pyroptosis and apoptosis. FASEB J..

[259] Zhou B., Abbott D.W. (2021). Gasdermin E permits interleukin-1 beta release in distinct sublytic and pyroptotic phases. Cell Rep..

[260] Tsuchiya K., Nakajima S., Hosojima S., Thi Nguyen D., Hattori T., Manh Le T., Hori O., Mahib M.R., Yamaguchi Y., Miura M. (2019). Caspase-1 initiates apoptosis in the absence of gasdermin D. Nat. Commun..

[261] Heilig R., Dilucca M., Boucher D., Chen K.W., Hancz D., Demarco B., Shkarina K., Broz P. (2020). Caspase-1 cleaves Bid to release mitochondrial SMAC and drive secondary necrosis in the absence of GSDMD. Life Sci. Alliance.

[262] Yoshitomi H. (2019). Regulation of Immune Responses and Chronic Inflammation by Fibroblast-Like Synoviocytes. Front. Immunol..

[263] Marsh L.J., Kemble S., Reis Nisa P., Singh R., Croft A.P. (2021). Fibroblast pathology in inflammatory joint disease. Immunol. Rev..

[264] Korb A., Pavenstadt H., Pap T. (2009). Cell death in rheumatoid arthritis. Apoptosis.

[265] Nygaard G., Firestein G.S. (2020). Restoring synovial homeostasis in rheumatoid arthritis by targeting fibroblast-like synoviocytes. Nat. Rev. Rheumatol..

[266] Izquierdo E., Canete J.D., Celis R., Del Rey M.J., Usategui A., Marsal S., Sanmarti R., Criado G., Pablos J.L. (2011). Synovial fibroblast hyperplasia in rheumatoid arthritis: Clinicopathologic correlations and partial reversal by anti-tumor necrosis factor therapy. Arthritis Rheum..

[267] Alivernini S., MacDonald L., Elmesmari A., Finlay S., Tolusso B., Gigante M.R., Petricca L., Di Mario C., Bui L., Perniola S. (2020). Distinct synovial tissue macrophage subsets regulate inflammation and remission in rheumatoid arthritis. Nat. Med..

[268] Jeong J.G., Kim J.M., Cho H., Hahn W., Yu S.S., Kim S. (2004). Effects of IL-1beta on gene expression in human rheumatoid synovial fibroblasts. Biochem. Biophys. Res. Commun..

[269] Ventura J.J., Cogswell P., Flavell R.A., Baldwin A.S., Davis R.J. (2004). JNK potentiates TNF-stimulated necrosis by increasing the production of cytotoxic reactive oxygen species. Genes Dev..

[270] Mueller M.B., Tuan R.S. (2011). Anabolic/Catabolic balance in pathogenesis of osteoarthritis: Identifying molecular targets. PM R.

[271] Buechler M.B., Fu W., Turley S.J. (2021). Fibroblast-macrophage reciprocal interactions in health, fibrosis, and cancer. Immunity.

[272] Bartok B., Firestein G.S. (2010). Fibroblast-like synoviocytes: Key effector cells in rheumatoid arthritis. Immunol. Rev..

[273] Scott B.B., Weisbrot L.M., Greenwood J.D., Bogoch E.R., Paige C.J., Keystone E.C. (1997). Rheumatoid arthritis synovial fibroblast and U937 macrophage/monocyte cell line interaction in cartilage degradation. Arthritis Rheum..

[274] Rannou F., Francois M., Corvol M.T., Berenbaum F. (2006). Cartilage breakdown in rheumatoid arthritis. Jt. Bone Spine.

[275] Miller M.C., Manning H.B., Jain A., Troeberg L., Dudhia J., Essex D., Sandison A., Seiki M., Nanchahal J., Nagase H. (2009). Membrane type 1 matrix metalloproteinase is a crucial promoter of synovial invasion in human rheumatoid arthritis. Arthritis Rheum..

[276] De Oliveira P.G., Farinon M., Sanchez-Lopez E., Miyamoto S., Guma M. (2019). Fibroblast-Like Synoviocytes Glucose Metabolism as a Therapeutic Target in Rheumatoid Arthritis. Front. Immunol..

[277] Friscic J., Bottcher M., Reinwald C., Bruns H., Wirth B., Popp S.J., Walker K.I., Ackermann J.A., Chen X., Turner J. (2021). The complement system drives local inflammatory tissue priming by metabolic reprogramming of synovial fibroblasts. Immunity.

[278] Zhang F., Wei K., Slowikowski K., Fonseka C.Y., Rao D.A., Kelly S., Goodman S.M., Tabechian D., Hughes L.B., Salomon-Escoto K. (2019). Defining inflammatory cell states in rheumatoid arthritis joint synovial tissues by integrating single-cell transcriptomics and mass cytometry. Nat. Immunol..

[279] Wei K., Korsunsky I., Marshall J.L., Gao A., Watts G.F.M., Major T., Croft A.P., Watts J., Blazar P.E., Lange J.K. (2020). Notch signalling drives synovial fibroblast identity and arthritis pathology. Nature.

[280] Stephenson W., Donlin L.T., Butler A., Rozo C., Bracken B., Rashidfarrokhi A., Goodman S.M., Ivashkiv L.B., Bykerk V.P., Orange D.E. (2018). Single-cell RNA-seq of rheumatoid arthritis synovial tissue using low-cost microfluidic instrumentation. Nat. Commun..

[281] Huang Q.Q., Doyle R., Chen S.Y., Sheng Q., Misharin A.V., Mao Q., Winter D.R., Pope R.M. (2021). Critical role of synovial tissue-resident macrophage niche in joint homeostasis and suppression of chronic inflammation. Sci. Adv..

[282] Kuo D., Ding J., Cohn I.S., Zhang F., Wei K., Rao D.A., Rozo C., Sokhi U.K., Shanaj S., Oliver D.J. (2019). HBEGF(+) macrophages in rheumatoid arthritis induce fibroblast invasiveness. Sci. Transl. Med..

[283] Zhou X., Franklin R.A., Adler M., Carter T.S., Condiff E., Adams T.S., Pope S.D., Philip N.H., Meizlish M.L., Kaminski N. (2022). Microenvironmental Sensing by Fibroblasts Controls Macrophage Population Size. bioRxiv.

[284] Zhou X., Franklin R.A., Adler M., Jacox J.B., Bailis W., Shyer J.A., Flavell R.A., Mayo A., Alon U., Medzhitov R. (2018). Circuit Design Features of a Stable Two-Cell System. Cell.

[285] Orr C., Vieira-Sousa E., Boyle D.L., Buch M.H., Buckley C.D., Canete J.D., Catrina A.I., Choy E.H.S., Emery P., Fearon U. (2017). Synovial tissue research: A state-of-the-art review. Nat. Rev. Rheumatol..

[286] Schonfeldova B., Zec K., Udalova I.A. (2021). Synovial single-cell heterogeneity, zonation, and interactions: A patchwork of effectors in arthritis. Rheumatology.

[287] Buckley C.D., Ospelt C., Gay S., Midwood K.S. (2021). Location, location, location: How the tissue microenvironment affects inflammation in RA. Nat. Rev. Rheumatol..

[288] Buechler M.B., Pradhan R.N., Krishnamurty A.T., Cox C., Calviello A.K., Wang A.W., Yang Y.A., Tam L., Caothien R., Roose-Girma M. (2021). Cross-tissue organization of the fibroblast lineage. Nature.

[289] Friedrich M., Pohin M., Jackson M.A., Korsunsky I., Bullers S.J., Rue-Albrecht K., Christoforidou Z., Sathananthan D., Thomas T., Ravindran R. (2021). IL-1-driven stromal-neutrophil interactions define a subset of patients with inflammatory bowel disease that does not respond to therapies. Nat. Med..

[290] Chu C.Q., Field M., Feldmann M., Maini R.N. (1991). Localization of tumor necrosis factor alpha in synovial tissues and at the cartilage-pannus junction in patients with rheumatoid arthritis. Arthritis Rheum..

[291] Udalova I.A., Mantovani A., Feldmann M. (2016). Macrophage heterogeneity in the context of rheumatoid arthritis. Nat. Rev. Rheumatol..

[292] Hatterer E., Shang L., Simonet P., Herren S., Daubeuf B., Teixeira S., Reilly J., Elson G., Nelson R., Gabay C. (2016). A specific anti-citrullinated protein antibody profile identifies a group of rheumatoid arthritis patients with a toll-like receptor 4-mediated disease. Arthritis Res. Ther..

[293] Kabala P.A., Angiolilli C., Yeremenko N., Grabiec A.M., Giovannone B., Pots D., Radstake T.R., Baeten D., Reedquist K.A. (2017). Endoplasmic reticulum stress cooperates with Toll-like receptor ligation in driving activation of rheumatoid arthritis fibroblast-like synoviocytes. Arthritis Res. Ther..

[294] Ospelt C., Brentano F., Rengel Y., Stanczyk J., Kolling C., Tak P.P., Gay R.E., Gay S., Kyburz D. (2008). Overexpression of toll-like receptors 3 and 4 in synovial tissue from patients with early rheumatoid arthritis: Toll-like receptor expression in early and longstanding arthritis. Arthritis Rheum..

[295] Midwood K., Sacre S., Piccinini A.M., Inglis J., Trebaul A., Chan E., Drexler S., Sofat N., Kashiwagi M., Orend G. (2009). Tenascin-C is an endogenous activator of Toll-like receptor 4 that is essential for maintaining inflammation in arthritic joint disease. Nat. Med..

[296] Kawaguchi M., Takahashi M., Hata T., Kashima Y., Usui F., Morimoto H., Izawa A., Takahashi Y., Masumoto J., Koyama J. (2011). Inflammasome activation of cardiac fibroblasts is essential for myocardial ischemia/reperfusion injury. Circulation.

[297] Sandanger O., Ranheim T., Vinge L.E., Bliksoen M., Alfsnes K., Finsen A.V., Dahl C.P., Askevold E.T., Florholmen G., Christensen G. (2013). The NLRP3 inflammasome is up-regulated in cardiac fibroblasts and mediates myocardial ischaemia-reperfusion injury. Cardiovasc. Res..

[298] Belibasakis G.N., Guggenheim B., Bostanci N. (2013). Down-regulation of NLRP3 inflammasome in gingival fibroblasts by subgingival biofilms: Involvement of Porphyromonas gingivalis. Innate Immun..

[299] Zhang A., Wang P., Ma X., Yin X., Li J., Wang H., Jiang W., Jia Q., Ni L. (2015). Mechanisms that lead to the regulation of NLRP3 inflammasome expression and activation in human dental pulp fibroblasts. Mol. Immunol..

[300] Bostanci N., Meier A., Guggenheim B., Belibasakis G.N. (2011). Regulation of NLRP3 and AIM2 inflammasome gene expression levels in gingival fibroblasts by oral biofilms. Cell Immunol..

[301] Ershaid N., Sharon Y., Doron H., Raz Y., Shani O., Cohen N., Monteran L., Leider-Trejo L., Ben-Shmuel A., Yassin M. (2019). NLRP3 inflammasome in fibroblasts links tissue damage with inflammation in breast cancer progression and metastasis. Nat. Commun..

[302] Kato M., Ospelt C., Gay R.E., Gay S., Klein K. (2014). Dual role of autophagy in stress-induced cell death in rheumatoid arthritis synovial fibroblasts. Arthritis Rheumatol..

[303] Shin Y.J., Han S.H., Kim D.S., Lee G.H., Yoo W.H., Kang Y.M., Choi J.Y., Lee Y.C., Park S.J., Jeong S.K. (2010). Autophagy induction and CHOP under-expression promotes survival of fibroblasts from rheumatoid arthritis patients under endoplasmic reticulum stress. Arthritis Res. Ther..

[304] Hong S., Kim E.J., Lee E.J., San Koo B., Min Ahn S., Bae S.H., Lim D.H., Kim Y.G., Yoo B., Lee C.K. (2015). TNF-alpha confers resistance to Fas-mediated apoptosis in rheumatoid arthritis through the induction of soluble Fas. Life Sci..

[305] Jaco I., Annibaldi A., Lalaoui N., Wilson R., Tenev T., Laurien L., Kim C., Jamal K., Wicky John S., Liccardi G. (2017). MK2 Phosphorylates RIPK1 to Prevent TNF-Induced Cell Death. Mol. Cell.

[306] Zhang D.W., Shao J., Lin J., Zhang N., Lu B.J., Lin S.C., Dong M.Q., Han J. (2009). RIP3, an energy metabolism regulator that switches TNF-induced cell death from apoptosis to necrosis. Science.

[307] Zhai Y., Wu B., Li J., Yao X.Y., Zhu P., Chen Z.N. (2016). CD147 promotes IKK/IkappaB/NF-kappaB pathway to resist TNF-induced apoptosis in rheumatoid arthritis synovial fibroblasts. J. Mol. Med..

[308] Zhang D.W., Zheng M., Zhao J., Li Y.Y., Huang Z., Li Z., Han J. (2011). Multiple death pathways in TNF-treated fibroblasts: RIP3- and RIP1-dependent and independent routes. Cell Res..

[309] Sosna J., Voigt S., Mathieu S., Lange A., Thon L., Davarnia P., Herdegen T., Linkermann A., Rittger A., Chan F.K. (2014). TNF-induced necroptosis and PARP-1-mediated necrosis represent distinct routes to programmed necrotic cell death. Cell Mol. Life Sci..

[310] Kim Y.S., Morgan M.J., Choksi S., Liu Z.G. (2007). TNF-induced activation of the Nox1 NADPH oxidase and its role in the induction of necrotic cell death. Mol. Cell.

[311] Los M., Mozoluk M., Ferrari D., Stepczynska A., Stroh C., Renz A., Herceg Z., Wang Z.Q., Schulze-Osthoff K. (2002). Activation and caspase-mediated inhibition of PARP: A molecular switch between fibroblast necrosis and apoptosis in death receptor signaling. Mol. Biol. Cell.

[312] Rickard J.A., Anderton H., Etemadi N., Nachbur U., Darding M., Peltzer N., Lalaoui N., Lawlor K.E., Vanyai H., Hall C. (2014). TNFR1-dependent cell death drives inflammation in Sharpin-deficient mice. Elife.

[313] Alikhani M., Alikhani Z., Raptis M., Graves D.T. (2004). TNF-alpha in vivo stimulates apoptosis in fibroblasts through caspase-8 activation and modulates the expression of pro-apoptotic genes. J. Cell Physiol..

[314] Liu W., Liu J., Wang W., Wang Y., Ouyang X. (2018). NLRP6 Induces Pyroptosis by Activation of Caspase-1 in Gingival Fibroblasts. J. Dent. Res..

[315] Zhaolin Z., Guohua L., Shiyuan W., Zuo W. (2019). Role of pyroptosis in cardiovascular disease. Cell Prolif..

[316] Wu T., Zhang X.P., Zhang Q., Zou Y.Y., Ma J.D., Chen L.F., Zou Y.W., Xue J.M., Ma R.F., Chen Z. (2021). Gasdermin-E Mediated Pyroptosis-A Novel Mechanism Regulating Migration, Invasion and Release of Inflammatory Cytokines in Rheumatoid Arthritis Fibroblast-like Synoviocytes. Front. Cell Dev. Biol..

[317] Rothlin C.V., Carrera-Silva E.A., Bosurgi L., Ghosh S. (2015). TAM receptor signaling in immune homeostasis. Annu. Rev. Immunol..

[318] Schneider C., King R.M., Philipson L. (1988). Genes specifically expressed at growth arrest of mammalian cells. Cell.

[319] Manfioletti G., Brancolini C., Avanzi G., Schneider C. (1993). The protein encoded by a growth arrest-specific gene (gas6) is a new member of the vitamin K-dependent proteins related to protein S, a negative coregulator in the blood coagulation cascade. Mol. Cell Biol..

[320] Varnum B.C., Young C., Elliott G., Garcia A., Bartley T.D., Fridell Y.W., Hunt R.W., Trail G., Clogston C., Toso R.J. (1995). Axl receptor tyrosine kinase stimulated by the vitamin K-dependent protein encoded by growth-arrest-specific gene 6. Nature.

[321] Godowski P.J., Mark M.R., Chen J., Sadick M.D., Raab H., Hammonds R.G. (1995). Reevaluation of the roles of protein S and Gas6 as ligands for the receptor tyrosine kinase Rse/Tyro 3. Cell.

[322] Stitt T.N., Conn G., Gore M., Lai C., Bruno J., Radziejewski C., Mattsson K., Fisher J., Gies D.R., Jones P.F. (1995). The anticoagulation factor protein S and its relative, Gas6, are ligands for the Tyro 3/Axl family of receptor tyrosine kinases. Cell.

[323] Nagata K., Ohashi K., Nakano T., Arita H., Zong C., Hanafusa H., Mizuno K. (1996). Identification of the product of growth arrest-specific gene 6 as a common ligand for Axl, Sky, and Mer receptor tyrosine kinases. J. Biol. Chem..

[324] Ohashi K., Nagata K., Toshima J., Nakano T., Arita H., Tsuda H., Suzuki K., Mizuno K. (1995). Stimulation of sky receptor tyrosine kinase by the product of growth arrest-specific gene 6. J. Biol. Chem..

[325] Chen J., Carey K., Godowski P.J. (1997). Identification of Gas6 as a ligand for Mer, a neural cell adhesion molecule related receptor tyrosine kinase implicated in cellular transformation. Oncogene.

[326] Bhattacharyya S., Zagorska A., Lew E.D., Shrestha B., Rothlin C.V., Naughton J., Diamond M.S., Lemke G., Young J.A. (2013). Enveloped viruses disable innate immune responses in dendritic cells by direct activation of TAM receptors. Cell Host Microbe.

[327] Rothlin C.V., Ghosh S., Zuniga E.I., Oldstone M.B., Lemke G. (2007). TAM receptors are pleiotropic inhibitors of the innate immune response. Cell.

[328] Deng T., Zhang Y., Chen Q., Yan K., Han D. (2012). Toll-like receptor-mediated inhibition of Gas6 and ProS expression facilitates inflammatory cytokine production in mouse macrophages. Immunology.

[329] Alciato F., Sainaghi P.P., Sola D., Castello L., Avanzi G.C. (2010). TNF-alpha, IL-6, and IL-1 expression is inhibited by GAS6 in monocytes/macrophages. J. Leukoc. Biol..

[330] Camenisch T.D., Koller B.H., Earp H.S., Matsushima G.K. (1999). A novel receptor tyrosine kinase, Mer, inhibits TNF-alpha production and lipopolysaccharide-induced endotoxic shock. J. Immunol..

[331] Lu Q., Lemke G. (2001). Homeostatic regulation of the immune system by receptor tyrosine kinases of the Tyro 3 family. Science.

[332] Wium M., Paccez J.D., Zerbini L.F. (2018). The Dual Role of TAM Receptors in Autoimmune Diseases and Cancer: An Overview. Cells.

[333] DeBerge M., Glinton K., Subramanian M., Wilsbacher L.D., Rothlin C.V., Tabas I., Thorp E.B. (2021). Macrophage AXL receptor tyrosine kinase inflames the heart after reperfused myocardial infarction. J. Clin. Investig..

[334] Howangyin K.Y., Zlatanova I., Pinto C., Ngkelo A., Cochain C., Rouanet M., Vilar J., Lemitre M., Stockmann C., Fleischmann B.K. (2016). Myeloid-Epithelial-Reproductive Receptor Tyrosine Kinase and Milk Fat Globule Epidermal Growth Factor 8 Coordinately Improve Remodeling After Myocardial Infarction via Local Delivery of Vascular Endothelial Growth Factor. Circulation.

[335] DeBerge M., Yeap X.Y., Dehn S., Zhang S., Grigoryeva L., Misener S., Procissi D., Zhou X., Lee D.C., Muller W.A. (2017). MerTK Cleavage on Resident Cardiac Macrophages Compromises Repair After Myocardial Ischemia Reperfusion Injury. Circ. Res..

[336] Han J., Bae J., Choi C.Y., Choi S.P., Kang H.S., Jo E.K., Park J., Lee Y.S., Moon H.S., Park C.G. (2016). Autophagy induced by AXL receptor tyrosine kinase alleviates acute liver injury via inhibition of NLRP3 inflammasome activation in mice. Autophagy.

[337] Du Y., Lu Z., Yang D., Wang D., Jiang L., Shen Y., Du Q., Yu W. (2021). MerTK inhibits the activation of the NLRP3 inflammasome after subarachnoid hemorrhage by inducing autophagy. Brain Res..

[338] Najafov A., Mookhtiar A.K., Luu H.S., Ordureau A., Pan H., Amin P.P., Li Y., Lu Q., Yuan J. (2019). TAM Kinases Promote Necroptosis by Regulating Oligomerization of MLKL. Mol. Cell.

[339] Newton K., Dugger D.L., Maltzman A., Greve J.M., Hedehus M., Martin-McNulty B., Carano R.A., Cao T.C., van Bruggen N., Bernstein L. (2016). RIPK3 deficiency or catalytically inactive RIPK1 provides greater benefit than MLKL deficiency in mouse models of inflammation and tissue injury. Cell Death Differ..

[340] Cai B., Thorp E.B., Doran A.C., Subramanian M., Sansbury B.E., Lin C.S., Spite M., Fredman G., Tabas I. (2016). MerTK cleavage limits proresolving mediator biosynthesis and exacerbates tissue inflammation. Proc. Natl. Acad. Sci. USA.

[341] Van den Brand B.T., Abdollahi-Roodsaz S., Vermeij E.A., Bennink M.B., Arntz O.J., Rothlin C.V., van den Berg W.B., van de Loo F.A. (2013). Therapeutic efficacy of Tyro3, Axl, and Mer tyrosine kinase agonists in collagen-induced arthritis. Arthritis Rheum..

[342] Waterborg C.E.J., Beermann S., Broeren M.G.A., Bennink M.B., Koenders M.I., van Lent P., van den Berg W.B., van der Kraan P.M., van de Loo F.A.J. (2018). Protective Role of the MER Tyrosine Kinase via Efferocytosis in Rheumatoid Arthritis Models. Front. Immunol..

[343] Biasizzo M., Kopitar-Jerala N. (2020). Interplay Between NLRP3 Inflammasome and Autophagy. Front. Immunol..

[344] Davidson S., Coles M., Thomas T., Kollias G., Ludewig B., Turley S., Brenner M., Buckley C.D. (2021). Fibroblasts as immune regulators in infection, inflammation and cancer. Nat. Rev. Immunol..

[345] Nagata S. (2018). Apoptosis and Clearance of Apoptotic Cells. Annu. Rev. Immunol..

[346] De Vasconcelos N.M., Van Opdenbosch N., Van Gorp H., Parthoens E., Lamkanfi M. (2019). Single-cell analysis of pyroptosis dynamics reveals conserved GSDMD-mediated subcellular events that precede plasma membrane rupture. Cell Death Differ..

[347] Li Z., Venegas V., Nagaoka Y., Morino E., Raghavan P., Audhya A., Nakanishi Y., Zhou Z. (2015). Necrotic Cells Actively Attract Phagocytes through the Collaborative Action of Two Distinct PS-Exposure Mechanisms. PLoS Genet..

